# Mitochondrial Ca^2+^ Overload Underlies Aβ Oligomers Neurotoxicity Providing an Unexpected Mechanism of Neuroprotection by NSAIDs

**DOI:** 10.1371/journal.pone.0002718

**Published:** 2008-07-23

**Authors:** Sara Sanz-Blasco, Ruth A. Valero, Ignacio Rodríguez-Crespo, Carlos Villalobos, Lucía Núñez

**Affiliations:** 1 Instituto de Biología y Genética Molecular (IBGM), Universidad de Valladolid and Consejo Superior de Investigaciones Científicas (CSIC), Valladolid, Spain; 2 Departamento de Bioquímica y Biología Molecular, Facultad de Ciencias Químicas, Universidad Complutense de Madrid, Madrid, Spain; University of Southern California, United States of America

## Abstract

Dysregulation of intracellular Ca^2+^ homeostasis may underlie amyloid β peptide (Aβ) toxicity in Alzheimer's Disease (AD) but the mechanism is unknown. In search for this mechanism we found that Aβ_1–42_ oligomers, the assembly state correlating best with cognitive decline in AD, but not Aβ fibrils, induce a massive entry of Ca^2+^ in neurons and promote mitochondrial Ca^2+^ overload as shown by bioluminescence imaging of targeted aequorin in individual neurons. Aβ oligomers induce also mitochondrial permeability transition, cytochrome c release, apoptosis and cell death. Mitochondrial depolarization prevents mitochondrial Ca^2+^ overload, cytochrome c release and cell death. In addition, we found that a series of non-steroidal anti-inflammatory drugs (NSAIDs) including salicylate, sulindac sulfide, indomethacin, ibuprofen and R-flurbiprofen depolarize mitochondria and inhibit mitochondrial Ca^2+^ overload, cytochrome c release and cell death induced by Aβ oligomers. Our results indicate that i) mitochondrial Ca^2+^ overload underlies the neurotoxicity induced by Aβ oligomers and ii) inhibition of mitochondrial Ca^2+^ overload provides a novel mechanism of neuroprotection by NSAIDs against Aβ oligomers and AD.

## Introduction

Alzheimer's Disease (AD) is a devastating neurodegenerative disorder due to a massive neuron dysfunction and loss related to development of senile plaques that are made of amyloid β peptide (Aβ), a cleavage product of the amyloid precursor protein. Early affected areas in the brain are the cortex and hippocampus. However, neuropathological studies show frequent and varied cerebellar changes in the late stages of the disease [1]. In fact, the cerebellum has been shown to be a unique organ in terms of AD manifestations because it is virtually free of neurofibrillary pathology but there is an strong correlation between cerebellar atrophy -with large cell death in the granular layer- and duration and stage of AD [1]. Although many *in vitro* studies have been carried out using cortical and hippocampal neurons, cerebellar granule cells have been also used for studies of Aβ neurotoxicity [2]–[4]. Several mechanisms have been envisioned. First, the “inflammatory hypothesis” proposes that Aβ may promote a damaging inflammation reaction. This view is supported among other evidence by the neuroprotection afforded by NSAIDs [5]. Second, Aβ promotes mitochondrial dysfunction and apoptosis and this toxicity contributes to AD [6] but the mechanism is unclear. Aβ may associate with mitochondrial membranes in mutant mice and patients with AD and mitochondria from mutant mice show lower levels of oxygen consumption and reduced respiratory complex-associated enzymatic activity suggesting that mitochondria-bound Aβ may impact on mitochondrial activity 7–9. Finally, AD has been also related to a general dyshomeostasis of intracellular Ca^2+^, a key second messenger involved in multiple neuronal functions. This view is supported by reports on dysregulation of intracellular Ca^2+^ promoted by Aβ and mutant presenilins [10]. Aβ may promote Ca^2+^ entry into neurons but results are controversial [11], [12]. Part of the controversy may relate to the fact that Aβ toxicity depends on its assembly state that varies from monomers to small, soluble oligomers and fibrils [13]. Small assemblies (oligomers) of unmodified Aβ are becoming the proximate neurotoxin in AD [13], [14], but most studies used fibrils. Intracellular Ca^2+^ levels are important for AD since overexpression of calbindin28k, an endogenous Ca^2+^ buffer, prevents neuron death in AD models [15]. However the link between putative changes in intracellular Ca^2+^ and cell damage is unknown. A rise in mitochondrial Ca^2+^ concentration ([Ca^2+^]_mit_) might contribute to neurotoxicity but monitoring [Ca^2+^]_mit_ in individual neurons has been challenging. We have addressed the effects of Aβ assembly state on Ca^2+^ influx and mitochondrial Ca^2+^ uptake using photon counting imaging of neurons expressing targeted aequorin [16]. We found that only oligomers, but not fibrils, increased cytosolic and mitochondrial Ca^2+^ concentrations. Accordingly we asked for the role of mitochondrial Ca^2+^ uptake on neurotoxicity induced by Aβ oligomers. Finally, we tested whether NSAIDs may protect against Aβ toxicity acting on subcellular Ca^2+^ fluxes. For these studies we have used mainly cerebellar granule cells although some experiments have been also carried out in cortical and hippocampal neurons.

## Results

### Aβ oligomers but not fibrils induce entry of Ca^2+^ into neurons

We have used the protocol reported by Klein [17] to prepare oligomers and fibrils from Aβ_1–42_ obtained from a commercial source (Bachem). Since the standard protocol of preparation includes a precipitation step in which some protein sample is lost, we hydrolyzed an aliquot of the final solution of both oligomers and fibrils in order to carry out an amino acid analysis. This procedure allowed us to obtain the real concentration of these compounds in solution. In the second place we characterized the quaternary structure (dimers, trimers, tetramers, etc.) of both the oligomers and fibrils using non-denaturing SDS-PAGE (pseudo-native gels). We were unable to stain the Aß_1–42_ peptides using Coomassie blue even when 2 µg of peptide were loaded per lane (data not shown). However, using silver staining we were able to determine the presence of high-molecular mass species in the SDS-PAGE. We rationalized that despite the fact that the samples were not boiled, a significant population of protein-protein interactions might be lost in the presence of SDS. However, were able to clearly identify monomers, dimers and tetramers in our preparation of oligomers (Figure S1A). This migration pattern of Aß_1–42_ oligomers in SDS-PAGE gels is well characterized [18]. When the preparation of fibrils was analyzed by SDS-GEL and silver staining, we could unambiguously identify the presence of monomers, dimers, trimers, tetramers and some larger oligomerization species in the gel. In addition, a certain amount of large molecular weight fibrils appeared at the top, incapable of entering the separating gel (Figure S1B). Similar results were obtained when the gel was transferred to a nitrocellulose membrane and the distribution of high-molecular mass species was determined by Western-blot using a monoclonal antibody raised against Aß_1–42_ (data not shown). In the third place, electron microscopy was used in order to characterize our Aß_1–42_ fibrils. Negative staining using uranyl acetate undoubtedly showed the presence of large fibrils in solution (Figure S1C). Most of these fibrils were similar in width and with a length that usually varied between 200 and 800 nm.

**Figure 1:**
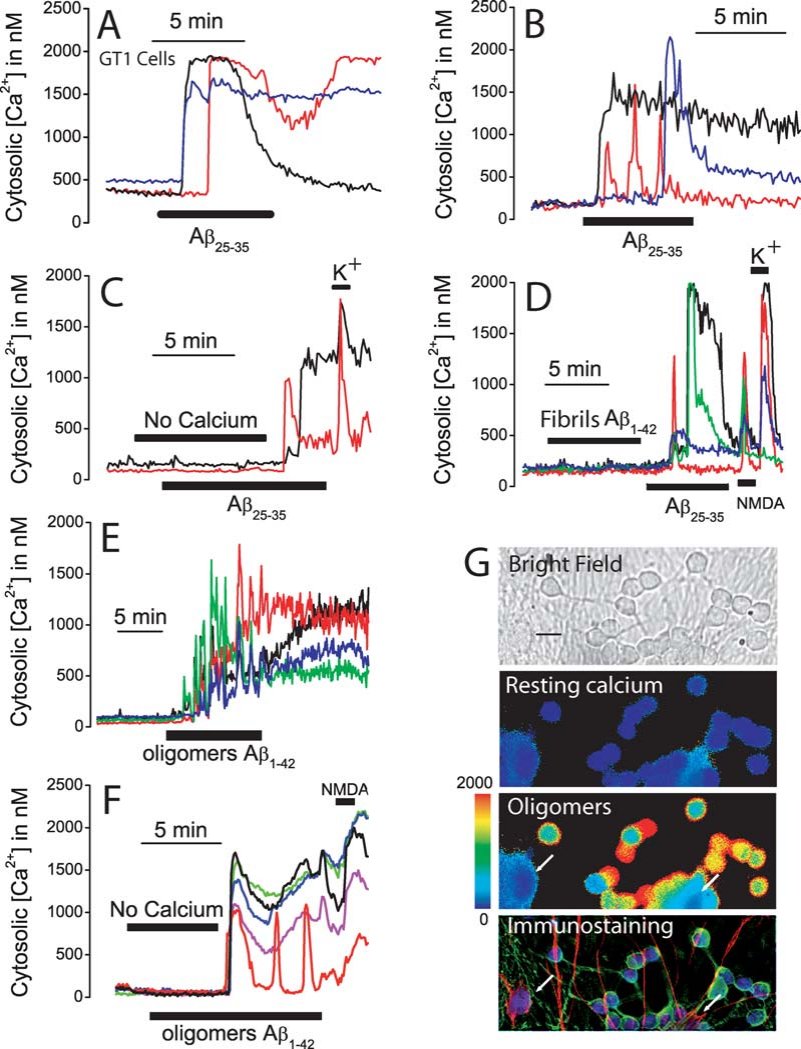
Aβ_1–42_ oligomers but not fibrils induce Ca^2+^ influx in neurons. *A–B*. GT1 neural cells and cerebellar granule cells were loaded with fura2/AM and subjected to calcium imaging. Traces show the effects of Aβ_25–35_ (20 µM) on [Ca^2+^]_cyt_ in three representative GT1 neural cells (*A*) and cerebellar granule cells (*B*). Data representative of 141–233 cells studied in 4 and 3 independent experiments, respectively. *C.* The increase in [Ca^2+^]_cyt_ induced by Aβ_25–35_ (20 µM) in cerebellar granule cells is abolished in medium lacking extracellular Ca^2+^ (No calcium). Addition of normal medium containing 1 mM Ca^2+^ restored the response to Aβ_25–35_. High K^+^ medium (150 mM K^+^) induced a further increase in [Ca^2+^]_cyt_. Traces correspond to two individual cells representative of n = 98 cells, 2 experiments). *D*. Aβ_1–42_ fibrils (2 µM) induced little or no [Ca^2+^]_cyt_ increase in cerebellar granule cells. The same cells responded Aβ_25–35_ (20 µM), N-methyl D-aspartate (100 µM NMDA) and high K^+^ medium (150 mM K^+^). (Traces correspond to four individual cells representative of n = 90 cells 3 experiments). *E*. Aβ_1–42_ oligomers (500 nM) induced a large and sustained increase in [Ca^2+^]_cyt_ in cerebellar granules cells. Traces correspond to four individual cells representative of n = 404 cells 5 experiments). *F*. The effects of Aβ_1–42_ oligomers (500 nM) are inhibited in medium lacking extracellular Ca^2+^ (No calcium). Perfusion of medium containing Ca^2+^ restored the response to Aβ oligomers. Cells also responded to 100 µM NMDA (recordings correspond to 5 individual cells representative of n = 283 cells, 3 experiments). *G*. Cerebellar granule cells were subjected to calcium imaging and then identified by double immunocytochemistry. Pictures show a bright field image (scale bar represents 10 µm) and [Ca^2+^]_cyt_ levels before (resting calcium) and after treatment with Aβ_1–42_ oligomers (oligomers) coded in pseudocolor (pseudocolor bar from 0 to 2000 nM shown at left) and immunostaining of the cells. Glial cells (arrows) are coded in red, neurons are coded in green and nuclei are coded in blue. Only neurons responded to Aβ oligomers with a rise in [Ca^2+^]_cyt_. Data representative of 249 cells, 3 experiments.

Once Aβ preparations were characterized, we studied the effects of oligomer and fibril preparations on [Ca^2+^]_cyt_. As a positive control, we used also the toxic fragment Aβ_25–35_ at large concentrations (20 µM) that have been shown previously to be neurotoxic and produce large increases in [Ca^2+^]_cyt_ in several neuron models including GT1 neural cells [19]. The effects of Aβ_1–42_ oligomers, Aβ_1–42_ fibrils and the toxic fragment Aβ_25–35_ (a surrogate of Aβ) on cytosolic Ca^2+^ concentration ([Ca^2+^]_cyt_) were tested by fluorescence microscopy (Fig. 1). We find that Aβ_25–35_ increases [Ca^2+^]_cyt_ in both GT1 neural cells (Fig. 1A) and cerebellar granule cells (Fig. 1B). In both cell types, increases in [Ca^2+^]_cyt_ are heterogeneous and show different lags and kinetics. In about two thirds of the cells, [Ca^2+^]_cyt_ remain increased even after washout of the peptide. The increase in [Ca^2+^]_cyt_ induced by Aβ_25–35_ is abolished in medium lacking extracellular Ca^2+^ and the responses resume after restoring extracellular Ca^2+^ (Fig. 1C) suggesting that Aβ_25–35_ promotes entry of Ca^2+^ rather than release from intracellular Ca^2+^ stores. Cerebellar granule cells responded also to high K^+^ medium (K) after Aβ_25–35_ (Fig. 1C). Next we tested the effects of Aβ_1–42_ in its different assembly states (fibrils and oligomers) prepared from synthetic Aβ_1–42_ [17], [20] in cerebellar granule cells. It has been reported that Aβ_1–42_ fibrils are less toxic than Aβ_1–42_ oligomers but produce the same toxicity when used at larger concentrations [13]. For example, at concentrations of about 5 µM, Aβ_1–42_ fibrils are equally toxic than Aβ_1–42_ oligomers at concentrations ranking 100 nM–1 µM. We found that, at 500 nM, Aβ_1–42_ fibrils induce no [Ca^2+^]_cyt_ increase (data not shown). At 2 µM, Aβ_1–42_ fibrils induce little or no [Ca^2+^]_cyt_ rise despite that Aβ_25–35_ increases [Ca^2+^]_cyt_ in the same neurons and cells responded also to N-methyl D-aspartate (NMDA) and K^+^ (Fig. 1D). In contrast to fibrils, Aβ_1–42_ oligomers, at 500 nM, induce a large and sustained increase in [Ca^2+^]_cyt_ in most cells (Fig. 1E). The increases in [Ca^2+^]_cyt_ induced by Aβ_1–42_ oligomers are heterogeneous showing different lags and kinetics and often [Ca^2+^]_cyt_ remain increased after washout of the peptide. The increase in [Ca^2+^]_cyt_ induced by Aβ_1–42_ oligomers is also inhibited in medium lacking extracellular Ca^2+^ (Fig. 1F) and addition of extracellular Ca^2+^ restores the response. Aβ_25–35_ and Aβ_1–42_ oligomers increase [Ca^2+^]_cyt_ in 90±9 and 82±9% of the cells, respectively. The maximum Δ[Ca^2+^]_cyt_ for responsive cells was 1290±247 nM for Aβ_25–35_ and 1050±63 nM for Aβ_1–42_ oligomers. In contrast, only 12±4% of the cells responded to Aβ_1–42_ fibrils and the maximum Δ[Ca^2+^]_cyt_ for responsive cells was 258±23 nM (data from 727 cells studied in 15 independent experiments). Most cells showing a Ca^2+^ response to Aβ_1–42_ oligomers also respond to NMDA suggesting that responsive cells are neurons rather than glia (Fig. 1F). To confirm this finding we identified responsive cells by means of two-fold immunocytochemistry against neurons and glia in the same microscopic field used for Ca^2+^ imaging. Fig. 1G shows a representative example in which cells were identified by immunostaining (red, glia; green, neurons; blue, nuclei) after Ca^2+^ imaging. Neurons but not glia undergo increases in [Ca^2+^]_cyt_ after presentation of Aβ_1–42_ oligomers.

**Figure 2:**
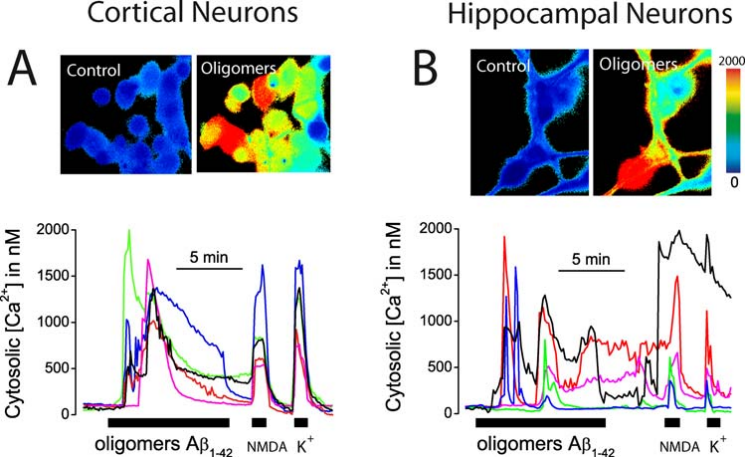
Aβ_1–42_ oligomers induce [Ca^2+^]_cyt_ increases in cortical and hippocampal neurons. Pictures show pseudocolor images of [Ca^2+^]_cyt_ before (control) and after (oligomers) perfusion with 500 nM Aβ_1–42_ oligomers in cortical (A) and hippocampal (B) neurons loaded with fura4F. Pseudocolor scale shown at right. Traces are recordings of [Ca^2+^]_cyt_ in 5 representative cortical (A) and hippocampal (B) neurons during stimulation with Aβ_1–42_ oligomers. Cells responding to Aβ_1–42_ oligomers also responded to 100 µM NMDA and 150 mM K^+^. Data are representative of n = 218 cortical cells and n = 149 hippocampal cells studied in at least 3 independent experiments for each brain area.

We have tested also the effects of Aβ_1–42_ oligomers on [Ca^2+^]_cyt_ in cortical and hippocampal neurons. Fig. 2 shows that Aβ_1–42_ oligomers (500 nM) induce very large increases in [Ca^2+^]_cyt_ in both cortical (Fig. 2A) and hippocampal neurons (Fig. 2B). Most cells present in the microscopic fields responded to Aβ_1–42_ oligomers in both cortical (83±9%, n = 218 cells, 3 experiments) and hippocampal (87±2%, n = 149 cells, 3 experiments) cells. In addition, the same cells that responded to Aβ_1–42_ oligomers also responded to NMDA suggesting that responsive cells are neurons rather than glia (Fig. 2). Taken together, the results indicate that Aβ_25–35_ and Aβ_1–42_ oligomers promote massive Ca^2+^ influx in GT1 neural cells, cerebellar granules as well as cortical and hippocampal neurons. In contrast, Aβ_1–42_ fibrils, at concentrations that are equally toxic than oligomers, produce no rises in [Ca^2+^]_cyt_ indicating that the mechanism of neurotoxicity may be different as proposed previously [21].

### Aβ oligomers induce mitochondrial Ca^2+^ overload

**Figure 3:**
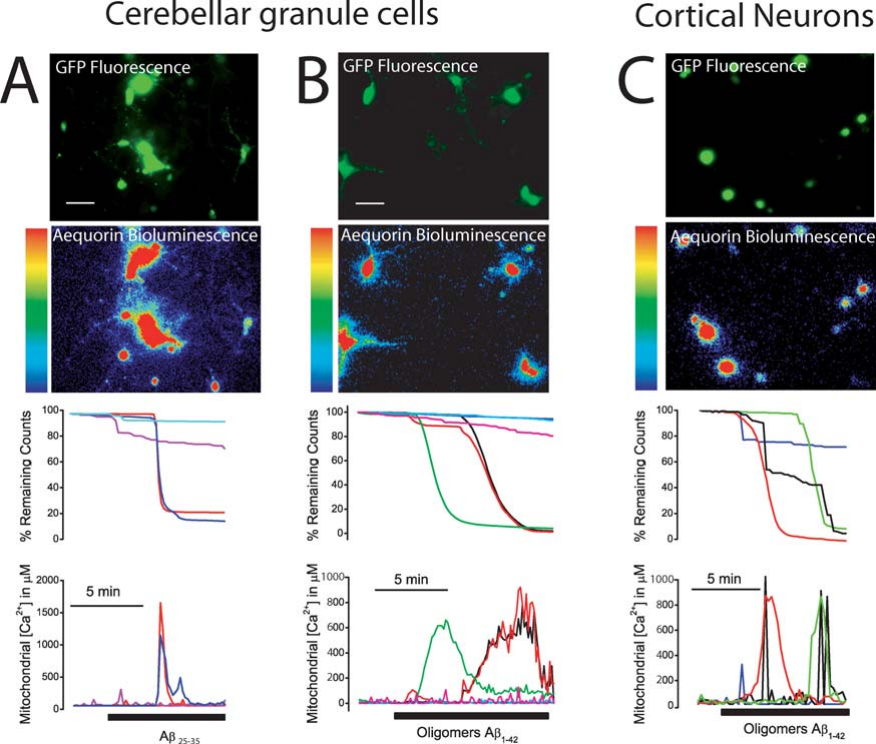
Aβ_1–42_ oligomers induce mitochondrial Ca^2+^ overload. Cerebellar granule cells and cortical neurons were transfected with the low-affinity, mitochondria-targeted aequorin fused to GFP, incubated with 1 µM n coelenterazine and subjected to bioluminescence imaging of [Ca^2+^]_mit_. Pictures show the fluorescence (top, GFP fluorescence) and accumulated photonic emissions (bottom, Aequorin Bioluminescence) images of representative microscopic fields (scale bar represents 10 µm). Luminescence intensity is coded in pseudocolor (1 to 32 photons per pixel). Traces show the effects of Aβ_25–35_ (20 µM, A) and Aβ_1–42_ oligomers (500 nM, B) on % of remaining counts (top traces) and calibrated [Ca^2+^]_mit_ (bottom traces) in individual, representative cerebellar granule cells (A,B) and cortical neurons (C). Data are representative of 37, 36 and 68 individual cells studied in 3 independent experiments, respectively.

We studied next whether Aβ peptides promote also mitochondrial Ca^2+^ uptake. We and others have reported previously that [Ca^2+^]_mit_ may rise substantially and that most [Ca^2+^]_mit_ measurements have been largely underestimated due to the use of non-targeted probes with high affinity for Ca^2+^ [22]–[24]. Accordingly, we used bioluminescence imaging of neurons expressing a low-affinity aequorin targeted to mitochondria developed previously to monitor the high-[Ca^2+^] levels inside the endoplasmic reticulum (ER, 24). This probe contains also GFP to select transfected neurons for bioluminescence imaging (mGA, 25). Fig. 3 shows the fluorescence (GFP, top) and bioluminescence (aequorin, bottom) images of cerebellar granule cells transfected with the mGA plasmid. We reported previously that stimulation of sympathetic neurons and other excitable cells expressing low-affinity, mitochondria-targeted aequorin with high K^+^ medium (to open voltage-gated Ca^2+^ channels) release typically 5–10% of photonic emissions (expressed as the % of remaining counts) that, when calibrated, reported increases in [Ca^2+^]_mit_ of about 100–300 µM. In addition, these [Ca^2+^]_mit_ rises were limited to a pool of mitochondria close to sites of Ca^2+^ entry and release [16], [23], [25]. We found that, in marked contrast, Aβ_25–35_ (Fig. 3A) and Aβ_1–42_ oligomers (Fig. 3B) released nearly all photonic emissions in responsive neurons which, after calibration, reported an average increase in [Ca^2+^]_mit_ close to the mM level. Like the rises in [Ca^2+^]_cyt_, the increases in [Ca^2+^]_mit_ were heterogeneous in terms of lag, size and kinetics. On average, Aβ_25–35_ induced a [Ca^2+^]_mit_ increase in 67±3% of the cells (mean±SEM, 1008±250 µM, 37 cells, 3 experiments) whereas 62±10% of the neurons responded to Aβ_1–42_ oligomers with a large increase in [Ca^2+^]_mit_ (960±200 µM, 36 cells, 3 experiments). In contrast, Aβ_1–42_ fibrils failed to induce any increase in [Ca^2+^]_mit_ (n = 3 experiments, data not shown) consistently with the lack of effects on [Ca^2+^]_cyt_. The rises in [Ca^2+^]_mit_ induced by Aβ_25–35_ and Aβ_1–42_ oligomers in intact neurons were similar to the increase in [Ca^2+^]_mit_ induced by perfusing permeabilized neurons with intracellular medium (see Methods) containing 5 µM Ca^2+^. This rise was prevented by the mitochondrial Ca^2+^ uniporter blocker ruthenium red and by collapsing the mitochondrial potential (ΔΨ) with 10 µM FCCP (data not shown). Aβ_25–35_ induced a similar large increase in both [Ca^2+^]_cyt_ and [Ca^2+^]_mit_ in GT1 neural cells (data not shown). We tested also the effects of Aβ_1–42_ on [Ca^2+^]_mit_ in cortical neurons. Aβ_1–42_ oligomers also induced large [Ca^2+^]_mit_ increases in a large population (75±5%, n = 91 cells, 6 experiments) of cortical neurons (Fig. 3C). In the responsive cortical neurons, the average rise in [Ca^2+^]_mit_ was 1099±93 µM (mean±SEM). Taken together, these results show that Aβ_25–35_ and Aβ_1–42_ oligomers, but not fibrils, induce a large and sustained entry of Ca^2+^ through the plasma membrane followed by mitochondrial Ca^2+^ overload in all neuronal cell models tested here. We asked next whether Aβ_1–42_ oligomers induce apoptosis and cell death and if so, for the possible role of mitochondrial Ca^2+^ overload in that process.

### Aβ induces apoptosis and cell death in neurons

**Figure 4:**
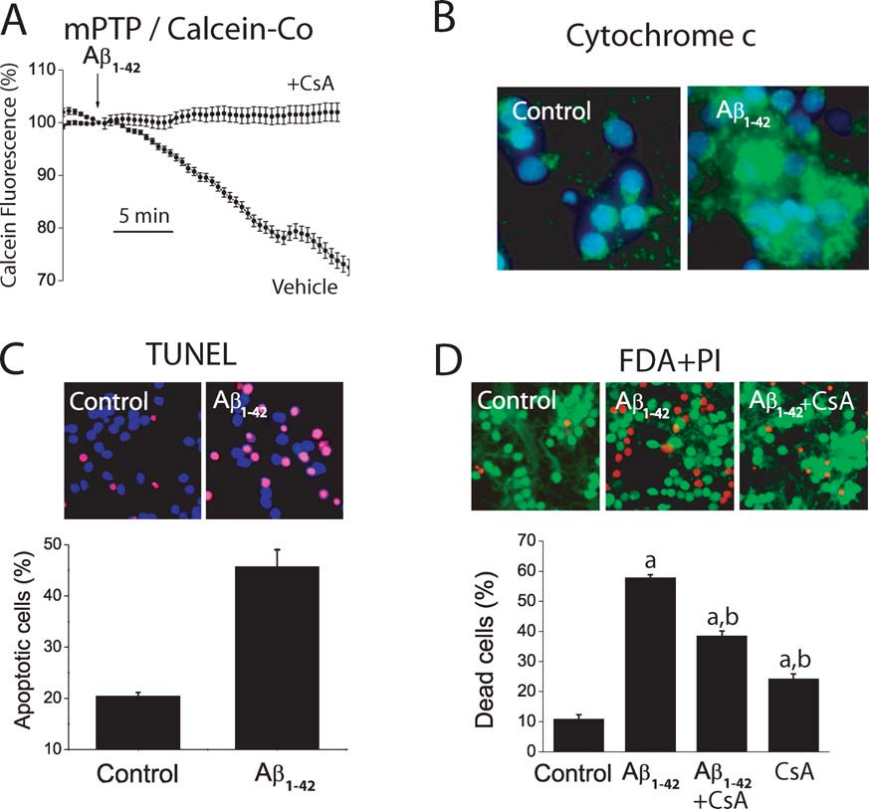
Aβ_1–42_ oligomers induce mPTP opening, cytochrome c release, apoptosis and cell death. *A*. Mitochondrial permeability transition was assessed directly by the calcein/Co^2+^ method. Cerebellar granule cells were loaded with calcein/AM 1 µM and CoCl_2_ 1 mM for 15 min at 37°C and calcein fluorescence quenching was imaged. Traces correspond to mean±SEM fluorescence records normalized to the value before the addition of oligomeric Aβ_1–42_ (500 nM) in responsive cells (n = 16 cells, vehicle) or in cyclosporin (1 µM) treated cells. Representative of 3 experiments. *B*. Location of cytochrome c was assessed by immunofluorescence against cytochrome c. Cerebellar granule cells were cultured for 72 h in vehicle or 500 nM Aβ_1–42_ oligomers. Blue colors show nuclei stained with DAPI. Green colors show location of cytochrome c. Control cells show punctate distribution of cytochrome c. Aβ oligomers-treated cells show a more diffuse location of cytochrome c due to release of cytochrome c from mitochondria. Representative of 3 independent experiments. Scale bar represents 10 µm. *C*. Cerebellar granule cells were cultured for 72 h in vehicle or Aβ_1–42_ oligomers (500 nM) and apoptosis was tested by TUNEL assay. Pictures show nuclei (blue) and apoptotic cells (purple). Bars show % of apoptotic cells. (n = 3; *p<0 05). Scale bar represents 10 µm. *D*. Cerebellar granule cells were cultured for 72 h with vehicle (control) or Aβ_1–42_ oligomers (500 nM) and cell death was assessed by staining with FDA (green living cells) and PI (red dead cells, scale bar represents 10 µm. Shown are also the effects cyclosporin A (1 µM) on Aβ_1–42_ oligomers-induced death in cerebellar granule cells (n = 3;^ a^p<0 05 vs control; ^b^p<0 05 vs Aβ_1–42_). Representative of 3 independent experiments.

Since mitochondrial Ca^2+^ overload may promote opening of the mitochondrial permeability transition pore (mPTP) and the intrinsic pathway to apoptosis [26], [27], we have studied whether exogenously added Aβ_1–42_ oligomers promote apoptotic processes in cerebellar granule cells. For assessing mPTP opening we used the calcein/Co^2+^ method [28]. Calcein fluorescence distributes in both cytosol and mitochondria but only cytosolic calcein is quenched by Co^2+^. Opening of mPTP promotes mitochondrial calcein quenching. Fig 4A shows that Aβ_1–42_ oligomers induce quenching of mitochondrial calcein fluorescence in cells loaded with calcein and CoCl_2_ and this effect is prevented by cyclosporin A, a mPTP blocker. Aβ_1–42_ oligomers also promote release of cytochrome c (Fig. 4B), apoptosis (Fig. 4C) and cell death (Fig. 4D) as shown by conventional immunofluorescence against cytochrome c, TUNEL assay and dye exclusion studies, respectively. Cell death induced by Aβ oligomers is inhibited by cyclosporin A (Fig. 4D). Aβ_25–35_ also induces apoptosis and cell death in cerebellar granule cells (Figure S2). These results indicate that Aβ_1–42_ oligomers promote a mitochondrial pathway to apoptosis. These effects are quite well mimicked by the toxic fragment Aβ_25–35_, although at far larger concentrations.

### Mitochondrial Ca^2+^ overload contributes to Aβ-induced apoptosis

**Figure 5:**
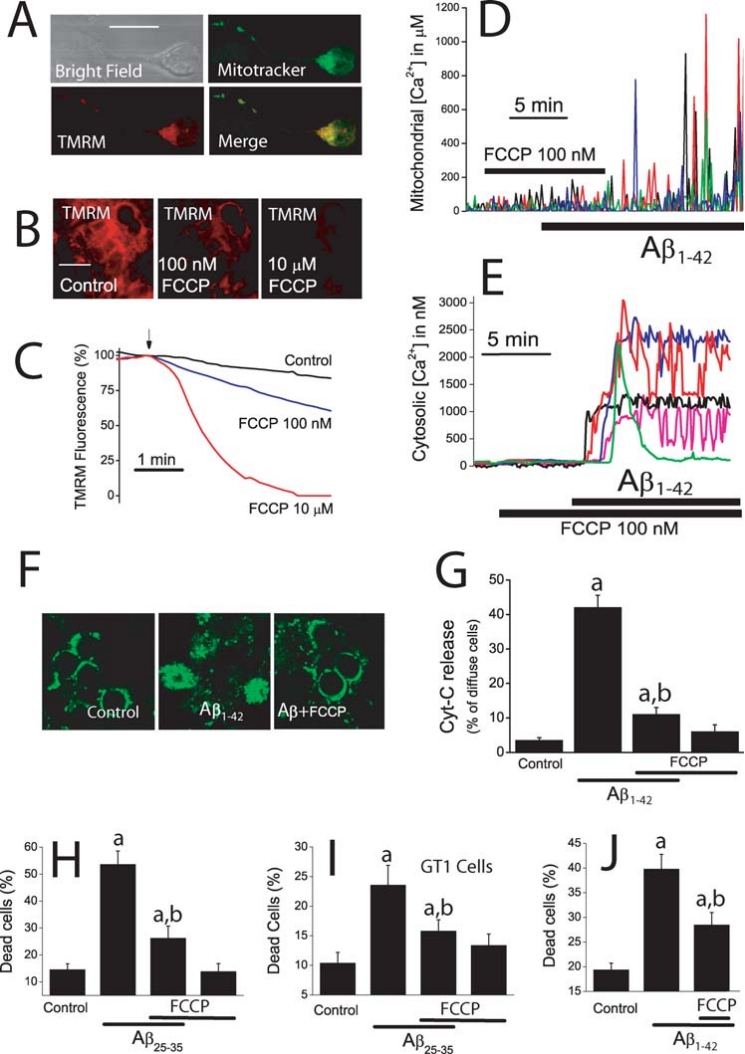
Mitochondrial Ca^2+^ uptake contributes to cell death induced by Aβ oligomers. *A*. Cerebellar granule cells were co-loaded with mitotracker green and TMRM 10 nM and washed. Double staining was assessed by confocal microscopy. Pictures show bright field mitotracker staining, TMRM staining and merge of mitotracker and TMRM staining. Images confirm the mitochondrial location of the TMRM probe. Scale bar represents 10 µm. *B*. Cells were loaded with TMRM 10 nM and mitochondrial depolarization was estimated by the decrease in TMRM fluorescence. Pictures show TMRM fluorescence images of cerebellar granule cells treated for 5 min with vehicle (control), 100 nM FCCP or 10 µM FCCP. Scale bar represents 10 µm. *C*. Traces show fluorescence recordings of cerebellar granule cells stained with 10 nM TMRM. Fluorescence values from individual cells were normalized to the value before addition (arrow) of either vehicle or FCCP and averaged. Each trace is the mean of 45–67 cells and representative of at least 3 independent experiments. *D*. Cerebellar granule cells expressing mGA were subjected to bioluminescence for [Ca^2+^]_mit_ measurements. FCCP 100 nM inhibits the increase in [Ca^2+^]_mit_ induced by Aβ_1–42_ oligomers (500 nM, 21 cells, 3 experiments). After washout of FCCP, Aβ_1–42_ oligomers were able to increase [Ca^2+^]_mit_. *E*. Cerebellar granule cells were loaded with fura2/AM and subjected to fluorescence imaging for [Ca^2+^]_cyt_ measurements. FCCP 100 nM failed to inhibit the increase in [Ca^2+^]_cyt_ induced by Aβ_1–42_ oligomers (500 nM) (n = 78 cells, 3 experiments). *F*. Immunofluorescence against cytochrome c was assessed by confocal microscopy in cerebellar granule cells treated with vehicle (Control), Aβ_1–42_ oligomers 500 nM (Aβ) and Aβ_1–42_ oligomers+FCCP 100 nM (Aβ+FCCP) for 72 h. Aβ_1–42_ oligomers promote diffusion of cytochrome c that otherwise shows a punctate staining. 100 nM FCCP prevented diffusion of cytochrome C. Scale bar represents 10 µm. *G*. Bars show % of cells showing diffuse staining for cytochrome c (reflecting cytochrome c release). Aβ_1–42_ oligomers (500 nM) increase the % of cells showing diffuse staining, an effect inhibited by FCCP 100 nM. *H–J*. FCCP 100 nM inhibits cell death induced by 20 µM Aβ_25–35_ in cerebellar cells (H) and GT1 cells (I) and by 500 nM Aβ_1–42_ oligomers in cerebellar granule cells (J) as assessed by dye exclusion studies. ^a^p<0 05 *vs*. control; All data are mean±SEM of 3 independent experiments.

We asked next for the contribution of mitochondrial Ca^2+^ overload to the apoptosis induced by Aβ_1–42_ oligomers and Aβ_25–35_. For this end we have studied whether inhibition of mitochondrial Ca^2+^ uptake prevents Aβ oligomers-induced apoptosis or not. Ca^2+^ uptake by mitochondria depends exponentially on ΔΨ, a huge driving force of −180 mV built upon the respiratory chain. We have reported recently that a small mitochondrial depolarization is enough to prevent largely mitochondrial Ca^2+^ uptake [29], [30]. Accordingly, we studied the effects of a small mitochondrial depolarization using low concentrations of the mitochondrial uncoupler FCCP. To assess the effects of this treatment on ΔΨ we used TMRM, a cationic dye that accumulates in mitochondria according to ΔΨ and is considered one of the most sensitive ΔΨ probes available [31]. Confocal images of cerebellar granule cells co-stained with mitotracker green and TMRM show the mitochondrial localization of the probe (Fig. 5A). Addition of FCCP decreases TMRM fluorescence in a dose–dependent manner consistent with a mitochondrial depolarization (Fig. 5B). At 100 nM, FCCP decreases TMRM fluorescence by about 25% relative to the total fluorescence decrease induced by 10 µM FCCP. According to our previous report [30], we estimate that such a decrease in TMRM fluorescence induced by 100 nM FCCP corresponds to a loss of ΔΨ of about 10–20 mV; whereas at 10 µM, FCCP decreases fully TMRM fluorescence consistently with a collapse of ΔΨ (Fig. 5C). Similar results were found in GT1 neural cells (data not shown). Strong plasma membrane depolarization with high K^+^ medium (50 mM) did not affect TMRM fluorescence excluding the possibility that TMRM is reporting changes in the plasma membrane potential (data not shown). Next, we studied the effects of such an small mitochondrial depolarization on mitochondrial Ca^2+^ uptake. We found that FCCP, at 100 nM, prevents the increase in [Ca^2+^]_mit_ induced by Aβ_1–42_ oligomers (Fig. 5D). Specifically, 100 nM FCCP inhibits by 78±4% the [Ca^2+^]_mit_ increase induced by Aβ_1–42_ oligomers (n = 21 cells, 3 experiments). FCCP also inhibited the rise in [Ca^2+^]_mit_ induced by Aβ_25–35_ (Figure S3A). Specifically, 100 nM FCCP inhibits by 82±5% the [Ca^2+^]_mit_ increase induced by Aβ_25–35_ (17 cells studied, 3 experiments). This effect is not due to inhibition of Ca^2+^ entry through the plasma membrane since 100 nM FCCP does not prevent at all the increase in [Ca^2+^]_cyt_ induced by Aβ_1–42_ oligomers (Fig. 5E) or Aβ_25–35_ (Figure S3B).

Once that conditions are established for specific inhibition of mitochondrial Ca^2+^ uptake, we addressed the contribution of mitochondrial Ca^2+^ overload to the cell death induced by Aβ_1–42_ oligomers. For this end, we studied the effects of 100 nM FCCP on Aβ_1–42_ oligomers-induced cytochrome c release and cell death. We find that FCCP at 100 nM prevents both cytochrome c release induced by both Aβ_1–42_ oligomers (Fig. 5F,G) and Aβ_25–35_ (Figure S3C,D). Consistently, we find that FCCP 100 nM inhibits cell death induced by both Aβ_25–35_ in cerebellar granule cells (Fig. 5H) and GT1 cells (Fig. 5I). Finally, 100 nM FCCP also inhibits cell death induced by Aβ_1–42_ oligomers in cerebellar granule cells (Fig. 5J). These results indicate that inhibition of mitochondrial Ca^2+^ overload by a partial mitochondrial depolarization protects against the neurotoxicity induced by Aβ_1–42_ oligomers and Aβ_25–35_.

It has been reported that mitochondrial Ca^2+^ overload during excitotoxicity may promote reactive oxygen species (ROS) production and contribute to neuron cell damage [32]. Accordingly, we also studied the effects of Aβ_1–42_ oligomers on ROS production in cerebellar granule cells. Incubation of cerebellar granule cells with 500 nM Aβ_1–42_ oligomers for 4 h elicited a clear increase in number of neurons strongly stained by the ROS probe CM-H2DCFDA. This effect was absent in cells incubated previously with 100 nM FCCP added here to prevent the Aβ_1–42_ induced increase in [Ca^2+^]_mit_ (Figure S4). These results suggest that mitochondrial Ca^2+^ overload may contribute to ROS production induced by Aβ_1–42_ oligomers.

### NSAIDs depolarize mitochondria and inhibit mitochondrial Ca^2+^ uptake

**Figure 6:**
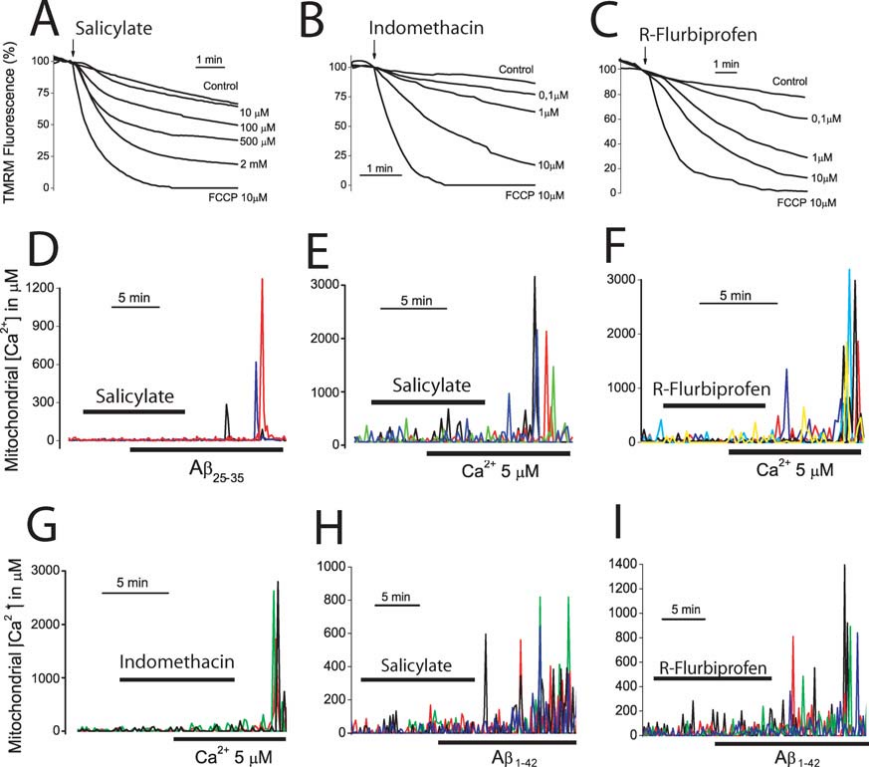
NSAIDs depolarize mitochondria and inhibit mitochondrial Ca^2+^ uptake. *A–C*. Cerebellar granule cells were stained with 10 nM TMRE and subjected to fluorescence microscopy for monitoring changes in mitochondrial potential. Each trace correspond to averaged, normalized fluorescence values of 34–42 cells treated (arrow) with vehicle (control) or different concentrations of salicylate (A, 10 µM to 2 mM), indomethacin (B, 0.1 to 10 µM), R-flurbiprofen (C, 0.1 to 10 µM). FCCP 10 µM was also added to compare with conditions of collapse of the mitochondrial potential. Each series of recordings is representative of at least 3 experiments. *D–F*. Cerebellar granule cells were transfected with the mGA plasmid and subjected to bioluminescence imaging for monitoring [Ca^2+^]_mit_. Salicylate (D, 100 µM) inhibits the [Ca^2+^]_mit_ induced by Aβ_25–35_ (20 µM, n = 43 cells, 3 experiments). In some experiments, cerebellar granule cells expressing mGA were permeabilized in intracellular medium containing 200 nM Ca^2+^ (see methods) and treated with salicylate 100 µM (E), R-flurbiprofen 1 µM (F) or indomethacin 1 µM (G) before being stimulated with the same intracellular medium containing 5 µM Ca^2+^ to stimulate mitochondrial Ca^2+^ uptake (Data from 188 cells studied in 6 independent experiments). *H,I.* Salicylate 100 µM and R-flurbiprofen 1 µM also inhibits the increase in [Ca^2+^]_mit_ induced by Aβ_1–42_ oligomers (500 nM). Data from n = 35 and 30 cells respectively, studied in 3 independent experiments for each drug.

**Figure 7:**
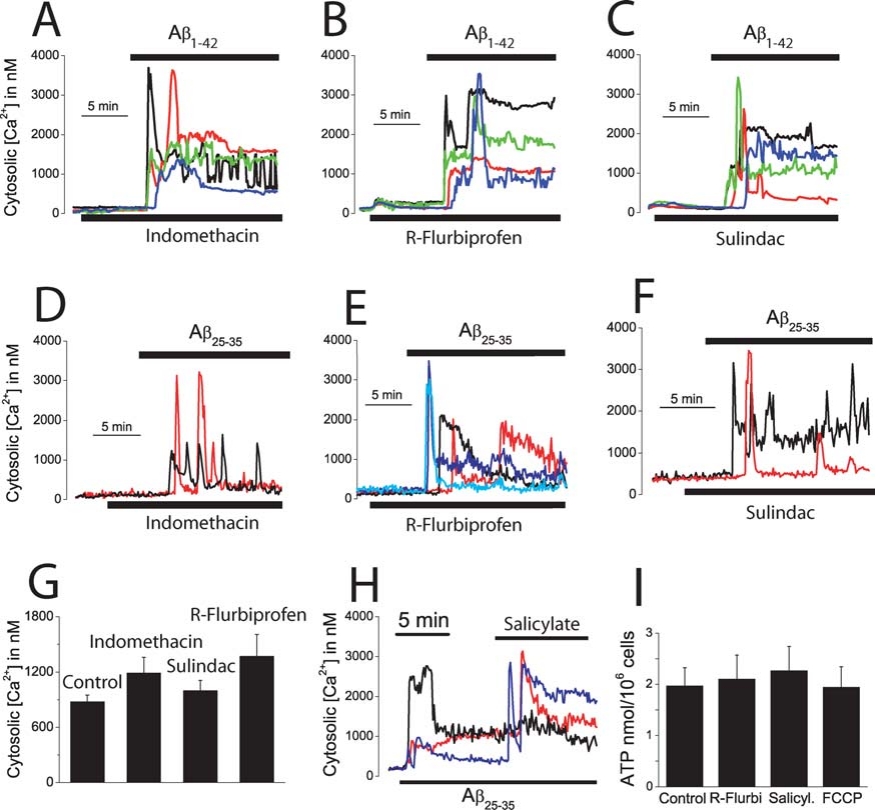
NSAIDs do not prevent the increase in [Ca^2+^]_cyt_ induced by Aβ_1–42-_ oligomers or affect cell ATP levels. *A–C*, cerebellar granule cells were loaded with fura4F/AM and subjected to calcium imaging to test the effects of several NSAIDs on the increases in [Ca^2+^]_cyt_ induced by Aβ_1–42_ oligomers. Neither indomethacin (A), R-flurbiprofen (B) or sulindac sulfide (C), all tested at 1 µM, inhibit the increase in [Ca^2+^]_cyt_ induced by Aβ_1–42_ oligomers. Data corresponds to 76–120 cells, corresponding to at least 2 experiments. *D–G,* indomethacin, R-flurbiprofen or sulindac sulfide (all tested at 1 µM) neither inhibit the increase in [Ca^2+^]_cyt_ induced by Aβ_25–35_. Shown are representative recordings of individual cells (46–90 cells studied in 3 independent experiments respectively). *G*. The maximum increase in [Ca^2+^]_cyt_ induced by Aβ_25–35_ (20 µM) was calculated for cells treated with vehicle (control) or the above NSAIDs (p>0,05; n = 3). *H*. Salicylate 100 µM increased [Ca^2+^]_cyt_ further in cells treated with Aβ_25–35_ (20 µM). Representative recordings of 164 cells studied in 3 independent experiments. *I*. Cerebellar granule cells were treated with vehicle, 1 µM R-flurbiprofen, 100 µM salicylate and 100 nM FCCP for 72 h and cell ATP levels were measured. None of the treatments decreases significantly cell ATP levels (p>0,05; n = 3).

The above results open the question on whether drugs preventing mitochondrial Ca^2+^ overload may or may not protect against Aβ_1–42_ oligomers and Aβ_25–35_. We have shown recently that salicylate, the mayor aspirin metabolite, depolarizes mitochondria and inhibit mitochondrial Ca^2+^ uptake in human colon carcinoma cells at low, therapeutic concentrations [29], [30]. Accordingly, we asked whether salicylate and other carboxylic NSAIDs depolarize mitochondria and inhibit mitochondrial Ca^2+^ uptake in cerebellar granule cells. Figs. 6A–C show that salicylate, indomethacin and R-flurbiprofen decrease TMRM fluorescence in a dose-dependent manner in intact neurons consistent with a partial mitochondrial depolarization. Shown are also the effects of 10 µM FCCP (lowest traces in each panel in Figs. 6A–C), a concentration that collapses the mitochondrial potential. A similar mitochondrial depolarization than that provoked by 100 nM FCCP (Fig. 5C) was elicited by 100 µM salicylate and by 0,1–1 µM of the remaining NSAIDs including R-flurbiprofen, an enantiomer derivative lacking anti-inflammatory activity. Next, we studied the effects of NSAIDs on mitochondrial Ca^2+^ uptake. We find that salicylate, indomethacin and R-flurbiprofen inhibit the mitochondrial Ca^2+^ overload induced by either Aβ_25–35_ in intact cells or by 5 µM Ca^2+^ in permeabilized neurons (Fig. 6D–G). Specifically, salicylate, at 100 µM, decreased by 78±4% the Aβ_25–35_-induced [Ca^2+^]_mit_ rise in intact cells and by 77±6% the Ca^2+^-induced rise in [Ca^2+^]_mit_ in permeabilized neurons (data from 157 cells studied in 4 independent experiments). Indomethacin, sulindac sulfide and R-flurbiprofen, all tested at 1 µM, inhibited by 77±3, 86±4 and 68±1%, respectively, the Ca^2+^-induced rise in [Ca^2+^]_mit_ in permeabilized neurons (data from 188 cells studied in 6 independent experiments). Salicylate and R-flurbiprofen also prevented the [Ca^2+^]_mit_ rise induced by Aβ_1–42_ oligomers (Fig. 6H,I). Specifically, salicylate at 100 µM and R-flurbiprofen at 1 µM inhibited by 77±4% and 85±3%, respectively (mean±SEM) the [Ca^2+^]_mit_ increase induced by Aβ_1–42_ oligomers (n = 35 and 30 cells, respectively, studied in 3 independent experiments for each drug). These results indicate that NSAIDs inhibit mitochondrial Ca^2+^ uptake induced by Aβ_1–42_ oligomers and Aβ_25–35_ in intact cells and by high Ca^2+^ in permeabilized cells. In addition, they suggest that this effect is probably secondary to mitochondrial depolarization and therefore, inhibition of the driving force for Ca^2+^ uptake through the mitochondrial Ca^2+^ uniporter, rather than inhibition of Ca^2+^ entry through the plasma membrane. To support further this view we assessed the effects of NSAIDs on the increases in [Ca^2+^]_cyt_ induced by Aβ_1–42_ oligomers and Aβ_25–35._ None of the NSAIDs tested including indomethacin, R-flurbiprofen and sulindac sulfide, all tested at 1 µM, produce any change in resting [Ca^2+^]_cyt_ or prevent the increase in [Ca^2+^]_cyt_ induced by Aβ_1–42_ oligomers (Fig. 7A–C) or Aβ_25–35_ (Fig. 7D–G). Moreover, addition of NSAID (salicylate) over the increased [Ca^2+^]_cyt_ induced by Aβ_25–35_ not only failed to decrease the level of [Ca^2+^]_cyt_ but rather increased it (Fig. 7H), an effect that can be explained by mitochondrial uncoupling and release of Ca^2+^ from preloaded mitochondria [33]. Because a large mitochondrial depolarization may affect cell metabolism, we tested whether or not the small depolarization induced by low concentrations of FCCP and NSAIDs may affect cell ATP levels. Fig. 7I shows that neither FCCP (100 nM), salicylate (100 µM) or R-flurbiprofen (1 µM) decrease the cell ATP levels in cerebellar granule cells. These results indicate that NSAIDs, at low µM concentrations, inhibit specifically mitochondrial Ca^2+^ uptake without preventing entry of Ca^2+^ through the plasma membrane and this effect is most likely mediated by a partial mitochondrial depolarization. We recently adapted an algorithm to convert TMR fluorescence values into estimated ΔΨ expressed in mV [30]. According to that algorithm, we estimate that the loss of ΔΨ induced by the low FCCP and NSAIDs concentrations used here is only 10–20 mV. This very small mitochondrial depolarization should not compromise cell metabolism as suggested by the lack of effects of these treatments on cell ATP levels.

### NSAIDs prevent neuron cell death induced by Aβ_1–42_ oligomers

**Figure 8:**
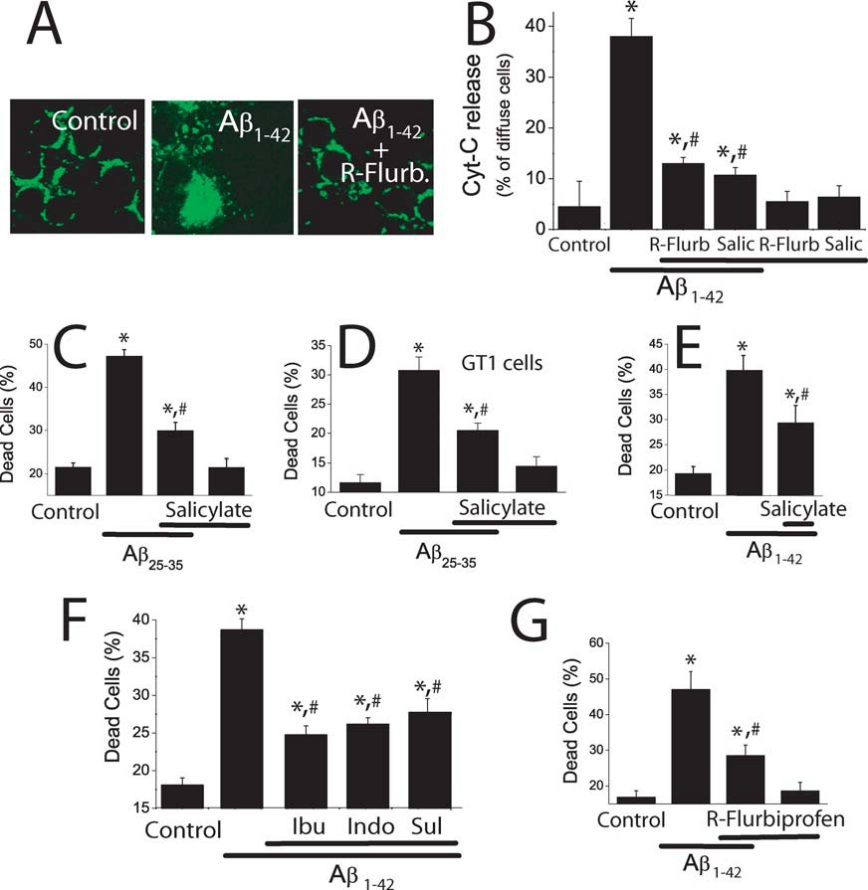
NSAIDs inhibit cytochrome c release and cell death induced by Aβ_1–42_ oligomers. *A,B*. Cerebellar granule cells were treated with Aβ_1–42_ (500 nM) for 72 h with or without 1 µM R-flurbiprofen or 100 µM salicylate and fixed for analysis of cytochrome c location using confocal microscopy. *A*. Control cells showed a punctate distribution of cytochrome c. Scale bar represents 10 µm. Aβ_1–42_ oligomers-treated cells show a more diffuse pattern of cytochrome c whereas cells treated with Aβ_1–42_ oligomers plus R-flurbiprofen show a punctate pattern similar to control cells. *B*. Bars show the relative abundance (%) of cells showing diffuse immunostaining in control cells, cells treated with Aβ_1–42_ oligomers and cells treated with Aβ_1–42_ oligomers plus 1 µM R-flurbiprofen or 100 µM salicylate (B). *p<0 05 vs control; #p<0 05 vs Aβ; data from at least 3 independent experiments. Salicylate 100 µM inhibits cell death induced by Aβ_25–35_ (20 µM) in cerebellar granule cells (C) and GT1 cells (D). Salicylate 100 µM also inhibits cell death induced by Aβ_1–42_ oligomers (500 nM) in cerebellar granule cells (E). (*p<0 05 vs control; #p<0 05 vs Aβ. n = 3). *F.* Ibuprofen (Ibu), Indomethacin (Indo), and sulindac sulfide (Sul), all tested at 1 µM, inhibit cell death induced by Aβ_1–42_ oligomers (500 nM) in cerebellar granule cells. *G.* R-Flurbiprofen 1 µM also inhibits cell death induced by Aβ_1–42_ oligomers (500 nM). *p<0 05 *vs*. control; ^#^p<0 05 *vs* Aβ. All data are representative of 3 experiments.

To learn whether inhibition of mitochondrial Ca^2+^ uptake by NSAIDs prevents cell death induced by Aβ_1–42_ oligomers, we have studied the effects of the same low NSAID concentrations on Aβ_1–42_ oligomers-induced cytochrome c release and cell death. We found that 1 µM R-flurbiprofen and 100 µM salicylate prevent cytochrome c release induced by Aβ_1–42_ oligomers (Fig. 8A,B). R-flurbiprofen and salicylate prevent also cytochrome c release induced by Aβ_25–35_ (Figure S5A–C). In addition, we found that 100 µM salicylate inhibits cell death induced by Aβ_25–35_ in both cerebellar granule cells (Fig. 8C) and GT1 cells (Fig. 8D). Salicylate also inhibits cell death induced by Aβ_1–42_ oligomers in cerebellar granule cells (Fig. 8E). Moreover, we found that 1 µM of either ibuprofen, indomethacin and sulindac sulfide prevented cerebellar granule cell death induced by Aβ_1–42_ oligomers (Fig. 8F) and Aβ_25–35_ (Figure S5D). R-flurbiprofen prevented cell death induced by either Aβ_1–42_ oligomers (Fig. 8G). In addition, R-flurbiprofen prevented cell death induced by Aβ_25–35_ to the same extent than the S enantiomer (Figure S5E). Thus, NSAIDs inhibit cytochrome c release and cell death induced by either Aβ_1–42_ and Aβ_25–35_ at the same low concentrations that depolarize partially mitochondria and inhibit mitochondrial Ca^2+^ overload. Finally, we found that 1 µM R-flurbiprofen, like 100 nM FCCP, also inhibits ROS production induced by Aβ_1–42_ oligomers (Figure S4).

## Discussion

We show here that Aβ_1–42_ oligomers, the assembly state that correlates best with brain damage and cognitive deficits in AD [13], [14], but not Aβ fibrils, promote massive entry of Ca^2+^ into GT1 neural cells and cerebellar granule neurons but not glia. This view is supported by the finding that cells responding to Aβ_1–42_ oligomers also respond to NMDA and by immunocytochemical identification of responsive cells. Aβ_1–42_ oligomers, but not fibrils, also induced large increases in [Ca^2+^]_cyt_ in cortical and hippocampal neurons. These results agree with those recently reported [34] showing that Aβ_1–42_ oligomers but not monomers or fibrils increased [Ca^2+^]_cyt_ in a human neuroblastoma cell line. The results suggest that the mechanism of neurotoxicity by fibrils and oligomers may be different as previously proposed [21]. The effects of Aβ_1–42_ oligomers are quite well reproduced by the toxic fragment Aβ_25–35_ although at 40-fold larger concentrations. The increases in [Ca^2+^]_cyt_ induced by Aβ_1–42_ oligomers and the toxic fragment Aβ_25–35_ can be attributed to enhanced entry of extracellular Ca^2+^ through the plasma membrane rather than release from intracellular Ca^2+^ stores as they were entirely prevented by removal of extracellular Ca^2+^. The route for this enhanced Ca^2+^ influx is not solved yet but candidate mechanisms may include plasma membrane permeabilization [34], formation of the so-called amyloid channels [35], [36] and/or activation of NMDA receptors [37]. Neither Aβ_25–35_ or Aβ_1–42_ oligomers induced parallel decreases in fura2 fluorescence excited at 340 and 380 nm (data not shown) suggesting that the rises in [Ca^2+^]_cyt_ are not due to membrane permeabilization. It has been shown previously that the increases in [Ca^2+^]_cyt_ induced by Aβ species can be prevented by the NMDA receptor open channel blocker memantine [37] and specific amyloid channel blockers [36] suggesting that both NMDA receptors and amyloid channels might be involved. In any case, the massive entry of Ca^2+^ induced by Aβ_1–42_ oligomers promotes mitochondrial Ca^2+^ overload as shown directly by bioluminescence imaging of neurons expressing a low-affinity aequorin targeted to mitochondria. This probe was originally developed to monitor the high [Ca^2+^] inside the endoplasmic reticulum that reach the mM range in resting conditions [24]. Using this probe, we and others have shown previously that [Ca^2+^]_mit_ may reach several hundred µM upon stimulation of voltage-gated Ca^2+^ entry and Ca^2+^ release from intracellular stores [16], [23], [24]. However, the above mentioned increases in [Ca^2+^]_mit_ were transient and restricted to a pool of mitochondria close to sites of Ca^2+^ entry or release [23], [24]. The recent introduction of low-affinity, targeted chameleons [38] has confirmed that [Ca^2+^]_mit_ may reach values above 200 µM, the sensitivity limit of these probes, stressing that [Ca^2+^]_mit_ levels actually rise substantially and the requirement of low-affinity probes for actual measurements of [Ca^2+^]_mit_, particularly when a mitochondrial Ca^2+^ overload is to be measured. Using the low-affinity, mitochondria-targeted aequorin, we find that Aβ_25–35_ and Aβ_1–42_ oligomers, but not fibrils, induce a massive mitochondrial Ca^2+^ overload that reaches values close to the mM level. As aequorin is consumed by the high Ca^2+^ level achieved, the results suggest that, at variance with stimulation with high K^+^, most mitochondria take up Ca^2+^ when cells are stimulated by Aβ_1–42_ oligomers. As mitochondrial Ca^2+^ uptake through the mitochondrial Ca^2+^ uniporter requires high [Ca^2+^]_cyt_ levels, these results suggest that Aβ_1–42_ oligomers promote a massive entry of Ca^2+^, large and sustained enough to activate the mitochondrial Ca^2+^ uniporter of most mitochondria.

**Figure 9:**
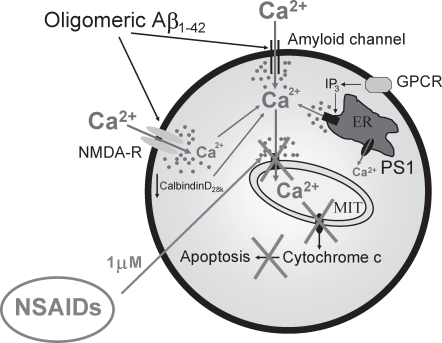
A model of Aβ-induced toxicity and neuroprotection by NSAIDs based on mitochondrial Ca^2+^. Aβ_1–42_ oligomers and the toxic fragment Aβ_25–35_ induce a large entry of Ca^2+^ through the plasma membrane likely mediated by formation of amyloid channels and/or NMDA receptors. This entry promotes in a sequential manner mitochondrial Ca^2+^ overload, ROS production, mPTP opening, cytochrome c release, apoptosis and cell death. Other factors related to AD may favor mitochondrial Ca^2+^ overload including exaggerated IP_3_-induced release of Ca^2+^ from the ER in loss of function PS1 mutants related to familial AD and/or decreased abundance of endogenous Ca^2+^ buffers as calbindinD28k during aging or sporadic AD. NSAIDs, at low concentrations (1 µM), depolarize partially mitochondria and inhibit mitochondrial Ca^2+^ overload, thus preventing cytochrome c release and apoptosis induced by Aβ oligomers.

It has been reported that mitochondrial Ca^2+^ overload may promote mPTP opening and apoptotic cell death [26], [39]. Our results indicate that mitochondrial Ca^2+^ overload contributes to the apoptotic cell death induced by Aβ oligomers. This view is supported by the findings that Aβ_1–42_ oligomers promote i)a mitochondrial Ca^2+^ overload in the whole mitochondrial population, ii)mitochondrial calcein quenching in a cyclosporin sensitive manner, iii)release of cytochrome c, iv)apoptosis as determined by TUNEL assay and v)cell death, again in a cyclosporin A-sensitive manner. Furthermore, inhibition of mitochondrial Ca^2+^ uptake by low concentrations of FCCP inhibit both cytochrome c release and cell death without preventing Aβ_1–42_ oligomers-induced increases in [Ca^2+^]_cyt_ or decreasing cell ATP levels. Finally, Aβ_1–42_ oligomers induce ROS production and this effect is prevented by low concentrations of FCCP. Taken together, our results suggest that the large and sustained entry of Ca^2+^ induced by Aβ_1–42_ oligomers, an effect mimicked by larger concentrations of the toxic fragment Aβ_25–35_, activate the mitochondrial Ca^2+^ uniporter of most mitochondria leading to a mitochondrial Ca^2+^ overload. This effect may promote mPTP opening by itself or, cooperate with the excess of ROS production promoted by the own mitochondrial Ca^2+^ overload. Finally, mPTP opening allows release of pro-apoptotic factors including cytochrome c leading to apoptosis and cell death (see proposed model in Fig. 9). This view resembles the mechanism of excitotoxicity reported for glutamate. In fact, glutamate-induced neuron death requires mitochondrial Ca^2+^ uptake [40]. In addition, low concentrations of FCCP have been reported to prevent mitochondrial Ca^2+^ uptake and cell death induced by NMDA [41]. Finally, glutamate induces also ROS production and this effect is prevented by mitochondrial uncoupling and blockers of the mitochondrial Ca^2+^ uniporter [32]. Whereas this mechanism may contribute largely to cell death induced by Aβ oligomers, our results do not exclude that additional mechanisms might contribute to this toxicity. For example, it has been reported that intracellular Aβ species may also interact with mitochondria in AD mouse models and affected AD brains [7]–[9] and this interaction may promote apoptosis and cell death. It remains to be established whether mitochondrial alterations induced by mitochondria-bound Aβ species cooperate with mitochondrial Ca^2+^ overload to promote cell death.

The above findings indicate that mitochondrial Ca^2+^ overload contributes to the neurotoxicity induced by Aβ_1–42_ oligomers. Accordingly, any strategy intended to prevent specifically mitochondrial Ca^2+^ uptake could potentially protect neurons against Aβ_1–42_ oligomers toxicity. Among the most promising agents for neuroprotection against AD are a series of classic NSAIDs [5], [42], [43]. Recent evidence indicates that neuroprotection afforded by NSAIDs is due to a mechanism other than their anti-inflammatory activity. This view is based in that some, but not all NSAIDs show neuroprotection and that structural analogs of classic NSAIDs lacking anti-inflammatory activity like, for instance, R-flurbiprofen, offer protection as well [43]. Some NSAIDs including R-flurbiprofen have been reported to target and inhibit γ-secretase activity at rather large concentrations (100 µM) leading to a lower Aβ burden [44], [45]. We show here that, at very low concentrations (1 µM), NSAIDs depolarize mitochondria to the same extent than low concentrations of FCCP, inhibit mitochondrial Ca^2+^ overload induced by Aβ oligomers without preventing Ca^2+^ entry through the plasma membrane, prevent ROS production induced by Aβ_1–42_ oligomers and inhibit Aβ oligomers-induced cytochrome c release and cell death. Accordingly we propose a novel mechanism of neuroprotection against soluble Aβ_1–42_ species by non-specific NSAIDs based on the primary inhibition of mitochondrial Ca^2+^ overload and prevention of the ensuing mPTP opening an downstream steps to cell death (Fig. 9). Notice that, at these low concentrations, NSAIDs have little or no effect on γ-secretase activity whereas fitting the concentration range achieved in brains by human therapeutic dose [45]. We must stress that these results have been obtained mainly in cerebellar granule cells, a brain area generally considered not affected earlier in AD. However, as mentioned above, neuropathological studies have shown frequent and varied cerebellar changes in the late stages of the disease [1]. Nevertheless, we show that Aβ_1–42_ oligomers also induced large increases in [Ca^2+^]_cyt_ and [Ca^2+^]_mit_ in cortical neurons as well as rises in [Ca^2+^]_cyt_ in hippocampal neurons.

The mechanism of inhibition of mitochondrial Ca^2+^ uptake by NSAIDs is most likely mediated by mitochondrial depolarization. This view is supported by i)the chemical structure of NSAIDs resembling mitochondrial uncouplers, ii)the release of Ca^2+^ induced by salicylate in Ca^2+^-overloaded cells and iii)direct TMRM fluorescence measurements showing NSAID-induced mitochondrial depolarization. In addition, uncoupling activity is considered a common characteristic of anti-inflammatory agents with an ionizable group [46]. It may seem surprising that a small mitochondrial depolarization be enough to inhibit largely mitochondrial Ca^2+^ uptake. However, several facts support this view. First the Ca^2+^ channel associated to the mitochondrial Ca^2+^ uniporter is inwardly rectifying, making it especially effective for Ca^2+^ uptake into energized mitochondria [47]. In this scenario, a mild mitochondrial depolarization will decrease largely the Ca^2+^ current through the mitochondrial Ca^2+^ uniporter. Second, the Nernst's equilibrium for Ca^2+^ across mitochondria predicts that a 50% fall in ΔΨ reduces the free mitochondrial [Ca^2+^] that can be reached at equilibrium by 1,000 fold [23]. This prediction is based on the huge ΔΨ built in respiring mitochondria and the fact that Ca^2+^ is a divalent cation present at extremely low concentrations (100 nM) in resting mitochondria. Thus, it is thermodynamically possible that even small drops in ΔΨ could influence dramatically the [Ca^2+^]_mit_ increase achieved during cell stimulation. In support of this view, we have shown recently that small drops in ΔΨ of a few tens of mVs are enough to prevent largely mitochondrial Ca^2+^ uptake [30].

Interestingly, other AD-related factors seemingly independent of Aβ species may contribute as well to mitochondrial Ca^2+^ overload and, perhaps, to neurotoxicity. For example, in familial AD, loss of function presenilin 1 mutants show exaggerated Ca^2+^ release from intracellular stores due to either defective Ca^2+^ leak from the ER or increased activity of Ca^2+^ release channels at ER [48–50, but see also 51 for alternative results]. The close coupling between ER and mitochondria [52] may favor mitochondrial Ca^2+^ overload in patients carrying these mutations. Further research is required to support this view. If that were the case, the possible PS1 mutation-mediated mitochondrial Ca^2+^ overload and ensuing neuron damage should be limited by NSAIDs. An even more important factor, at least from the point of view of the potential number of patients affected, could be the reported depletion of endogenous Ca^2+^ buffers, particularly calbindinD28k, in selected brain regions during aging and sporadic AD [53], [54]. A diminished cytosolic Ca^2+^ buffer capacity may place mitochondria at risk of Ca^2+^ overload during normal neural activity, a process that should be ameliorated by NSAIDs. Therefore, our results point to a pivotal role of mitochondrial Ca^2+^ overload in Aβ_1–42_ oligomers toxicity and AD. Interestingly, the mitochondrial Ca^2+^ overloads likely induced by AD related processes such as Aβ oligomers (shown here), excitotoxicity [40], excess Ca^2+^ release from ER [49] and loss of endogenous Ca^2+^ buffers [53], [54] could be all ameliorated by low NSAID concentrations, regardless of the source of Ca^2+^ excess being either intracellular or extracellular.

## Materials and Methods

### Materials

Wistar rats were obtained from the Valladolid University animal facility. Fura2/AM, TMRM, DAPI, CM-H2DCFDA, n coelenterazine, the cytochrome c antibody, Alexa 488 α mouse IgG and Alexa F594 α rabbit IgG were purchased from Invitrogen (Barcelona, Spain). DMEM (ref. 41966-029), fetal bovine serum, horse serum, neurobasal medium, B27, penicillin and streptomycin are from Gibco (Barcelona, Spain). Papain solution is from Worthington (Lakewood, NJ). The kit for TUNEL assay is from Roche Diagnostics (Penzberg, Germany). Aβ peptides were purchased from Bachem AG (Bubendorf, Switzerland). The mouse anti β-tubulin III is from Covance (Princeton, USA) and the rabbit anti-GFAP is from Sigma (Madrid, Spain). The mGA plasmid was kindly donated by P. Brulet (Gif-sur-Yvette, France). Hypothalamic GT1 neural cells were provided by R. Weiner (San Francisco, USA). Other reagents and chemicals were obtained either from Sigma or Merck.

### Cell Cultures

Cerebellar granule cells were obtained from 5-day old Wistar rat pups killed by dislocation followed by decapitation as reported previously [55]. Cortical neurons were obtained from P0 Wistar rat pups following the protocol reported by Murphy *et al.* [56]. Hippocampal neurons were prepared from P0 Wistar rat pups as reported by Brewer *et al.* [57] with the modifications introduced by Perez-Otano *et al*. [58]. Briefly, after brain removal, meninges were discarded and the hippocampus was separated from cortex. Hippocampal tissue was cut in small pieces, transferred to papain solution and incubated at 37° C for 30 minutes with occasional gentle shaking. Tissue pieces were washed with neurobasal medium and dissociated into a single cell suspension. Hippocampal cells were plated onto collagen-coated, 12 mm diameter glass coverslips at 40×10^3^ cells/dish, and grown in Neurobasal Medium supplemented with B27 and 10% horse serum. Cells were cultured for 7–10 days before experiments. Cerebellar granule cells and cortical neurons were plated on poly-L-lysine coated, 12 mm diameter glass coverslips and cultured in high-glucose, low K^+^, Dulbeccós modified Eaglés medium (DMEM, ref. 41966-029. Gibco, Spain) plus 10% fetal bovine serum, 5% horse serum, 100 u ml^−1^ penicillin and 100 µg ml^−1^ streptomycin for 2 days. Then the culture medium was replaced by Satós medium [59] plus 5% horse serum to avoid excessive proliferation of glia and cultured for 2–4 (cerebellar) or 3–5 (cortical) days before experiments. GT1 neural cells were grown in DMEM with 10% fetal bovine serum, 5% horse serum, 100 u ml^−1^ penicillin and 100 µg ml^−1^ streptomycin.

### Preparation of Aβ oligomers and fibrils

Aβ_1–42_ oligomers and fibrils were prepared as reported previously by Klein and Dahlgren et al. [17], [20]. Briefly, Aβ_1–42_ was initially dissolved to 1 mM in hexafluoroisopropanol and separated into aliquots in sterile microcentrifuge tubes. Hexafluoroisopropanol was removed under vacuum in a speed vac., and the peptide film was stored desiccated at −20°C. For the aggregation protocol, the peptide was first resuspended in dry dimethyl sulfoxide to a concentration of 5 mM and treated differently for preparation of oligomers and fibrils. For preparation of oligomers, Haḿs F-12 was added to bring the peptide to a final concentration of 100 µM and incubated at 4°C for 24 h. The preparation was then centrifuged at 14,000×g for 10 min at 4°C to remove insoluble aggregates (protofibrils and fibrils) and the supernatants containing soluble Aβ_1–42_ oligomers were transferred to clean tubes and stored at 4°C. For preparation of fibrils, the peptide resuspended in dimethyl sulfoxide at 5 mM concentration was diluted in 10 mM HCl to bring the peptide to a final concentration of 100 µM and incubated at 37°C for 24 h. Actual concentrations of both oligomers and fibrils were measured by amino acid analysis. Aβ_25–35_ was solved in PBS at a concentration of 1 mM, sonicated 3 times and stored at −20°C. For experiments, Aβ_25–35_ was solved in medium at a final concentration of 20 µM.

### Amino Acid analysis

Samples of both Aß_1–42_ oligomers and fibrils in glass tubes were extensively vacuum dried in a speed-vac. Hydrolysis was performed using a 5.9 HCl solution containing 0.1% phenol. Norleucine was added to the hydrolytic solution as an internal standard. The tubes were sealed under vacuum and the hydrolysis was performed during 24 h at 110°C. Subsequently, the samples were vacuum dried. Finally, the samples were injected into a Biochrom 30 amino acid analyzer. Both composition and concentration data was obtained from the chromatogram.

### PAGE-SDS and silver staining

A standard 17% PAGE-SDS was prepared and 10 µl samples of either Aβ_1–42_ oligomers or fibrils at a 40 µM concentration were incubated with 1X loading buffer. The samples were loaded in the gel without boiling and the gel was run at constant amperage of 20 mA. The gels were subsequently fixed with a freshly prepared solution of 50% methanol, 12% acetic acid, 0.02% paraformaldehyde for at least 1h. Then, the gel was washed three times with 50% ethanol. The next step consisted on an incubation of the gel for 1 min in 0.2 mg/ml NaS_2_O_3_. Subsequently, the gel was washed three times with miliQ water for 20 seconds. Then, the gel was incubated for 20 minutes in 2 mg/ml AgNO_3_ in 0.025% formaldehyde. After two 20-second washes with miliQ water, color was allowed to develop using a solution consisting of 60 mg/ml Na_2_CO_3_, 0.02% formaldehyde, 0.004 mg/ml NaS_2_O_3_. After the bands appear, the gel was washed twice with miliQ water and the reaction was stopped using a 50% methanol, 12% acetic acid solution.

### Electron microscopy

A 10 µl sample of Aß_1–42_ fibrils (40 µM) was applied to a 200 mesh Formvar-coated copper grid and incubated for 15 minutes at room temperature. The sample was then wicked off with filter paper, washed briefly by placing the grid face down on a droplet of water, and stained by transferring the grid face down on a droplet of 2% uranyl acetate for 2 minutes before wicking off the solution and air drying. Grids were visualized in a JEOL transmission electron microscope.

### Fluorescence imaging of [Ca^2+^]_cyt_ and in situ immunofluorescence

Coverslips containing cells were incubated in standard medium containing (in mM) NaCl 145, KCl 5, CaCl_2_ 1, MgCl_2_ 1, glucose 10 and Hepes 10 (pH, 7.42) and loaded with 4 µM fura2/AM or fura4F/AM for 60 min at room temperature. Then coverslips were placed on the heated stage of an inverted microscope (Nikon Diaphot), perfused continuously with the same prewarmed standard medium containing and epi-illuminated alternately at 340 and 380 nm. Light emitted above 520 nm was recorded with a Magical Image Processor (Applied Imaging). Pixel by pixel ratios of consecutive frames were captured and [Ca^2+^]_cyt_ was estimated from these ratios as previously reported [16]. For differential identification of neurons and glia, the single cell content of β-tubulin III and glial fibrillary acidic protein (GFAP) were assessed by indirect inmunofluorescence in the same cells used for calcium imaging as reported previously [55]. Briefly, after calcium imaging, cells were fixed with p-formaldehyde and incubated with anti β tubulin III (1∶400) and anti GFAP (1∶200) for 1 h at 37°C. Then, cells were washed and incubated with 1∶100 labeled anti IgG antibodies. Nuclei were stained by incubation with DAPI 0,2 µg/ml for 5 min.

### Bioluminescence imaging of [Ca^2+^]_mit_

Cells were transfected with the mGA plasmid using a Nucleofector II^®^ device and the VPG-1003 transfection kit (Amaxa Biosystems, Cologne, Germany). The mGA probe contain a mutated, low affinity aequorin targeted to mitochondria and a GFP sequence to help select transfected neurons [25]. After 24 h, cells were incubated for 1 h with 1 µM n coelenterazine, washed and placed into a perfusion chamber thermostated to 37 ^°^C under a Zeiss Axiovert S100 TV microscope and perfused at 5–10 ml/min with test solutions based on the standard perfusing solution described above prewarmed at 37°C. At the end of each experiment, cells were permeabilized with 0.1 mM digitonin in 10 mM CaCl_2_ to release all the residual aequorin counts. Bioluminescence images were taken with a Hamamatsu VIM photon counting camera handled with an Argus-20 image processor. Photonic emissions were integrated for 10 s periods. Photons/cell in each image were quantified using the Hamamatsu Aquacosmos software. Total counts per cell ranged between 2·10^3^ and 2·10^5^ and noise was (mean±SD) 1±1 counts per second (c.p.s.) per typical cell area (2000 pixels). Data were first quantified as rates of photoluminescence emission/total c.p.s remaining at each time (% of remaining counts) and divided by the integration period (L/L_TOTAL_ in s^−1^). Emission values of less than 4 c.p.s were not used for calculations. Calibrations in terms of [Ca^2+^]_mit_ were performed using as reported previously [23]. In some experiments, cells were permeabilized with digitonin 20 µM in “intracellular” medium of the following composition 130 mM KCl, 10 mM NaCl, 1 mM MgCl_2_, 1 mM K_3_PO_4_, 0,2 mM EGTA, 1 mM ATP, 20 µM ADP, 2 mM succinate, 20 mM HEPES/KOH, pH, 6.8. Then, the cells were incubated with the same medium containing 200 nM Ca^2+^ (buffered with EGTA) with or without NSAIDs for 5 min. Then, perfusion was switched to “intracellular” medium containing 5 µM Ca^2+^ (with or without NSAID). Further details have been reported previously [16], [23], [25].

### Mitochondrial permeability transition pore (mPTP)

mPTP opening was assessed directly by the calcein/cobalt method [28]. Cells were co-loaded with calcein-AM 1 µM and CoCl_2_ 1 mM for 30 min at 37°C and subjected to conventional fluorescence imaging or confocal microcopy. Fluorescence traces from individual cells were normalized relative to the value before addition of test solutions and averaged. Background fluorescence corresponding to regions of interest devoid of cells was subtracted. In some experiments, cells were incubated with cyclosporin A 1 µM for 15 min before recordings.

### ROS Formation

ROS formation was evaluated in live neurons using CM-H2DCFDA as reported by De Felice *et al*. [37]. Cerebellar granule cell cultures were incubated for 4 h at 37°C with vehicle or 500 nM Aβ_1–42_ in the absence or in the presence of FCCP 100 nM and R-flurbiprofen 1 µM. ROS formation was assessed using 2 µM CM-H2DCFDA with 40 min of probe loading. Then neurons were superfused for 5 min with prewarmed (37°C) PBS. Fluorescence in cells was immediately visualized using the Zeiss S100 TV inverted microscope, a FITC filter set, an OrcaER digital camera from Hamamatsu and the Hamamatsu Aquacosmos software.

### Mitochondrial potential (ΔΨ)

The effects of treatments on ΔΨ were estimated by fluorescence imaging in cells loaded with the ΔΨ sensitive probe TMRM as reported previously [29]–[31]. Briefly, cells were loaded with TMRM (10 nM) for 30 min at room temperature, placed on the perfusion chamber of a Zeiss Axiovert S100 TV inverted microscope and superfused continuously with the prewarmed (37°C) standard medium described above. Fluorescence images were taken at 10 s intervals with a Hamamatsu VIM photon counting camera handled with an Argus-20 image processor. Traces from individual cells were normalized relative to the value before the addition of either vehicle or treatment and averaged. Background fluorescence -after collapse of the mitochondrial potential induced by 10 µM FCCP- was subtracted. Location of TMRM staining and fluorescence intensity ratios of TMRM in mitochondrial and cytosolic areas was tested by confocal microcopy in cells stained with both TMRM and mitotracker green.

### Cytochrome c release

Cytochrome c release from mitochondria was tested by immunofluorescence and conventional or confocal microscopy. Cells were treated under the various experimental conditions for 72 h and fixed. Location of cytochrome c was tested by indirect immunofluorescence. In conventional fluorescence, nuclei were identified by DAPI staining. Confocal images were obtained using a BIO-RAD laser scanning system (Radiance 2100) coupled to a Nikon eclipse TE2100U, inverted microscope. For quantification of cytochrome c release by confocal microscopy, the relative abundance (%) of cells showing diffuse vs. punctate immunofluorescence was calculated [60].

### Cell death and apoptosis

Cells were plated in wells at about 5×10^4^ cells/ml and treated with test solutions for 72 h. Cell death was estimated in the same samples by staining with fluorescein diacetate (FDA, 50 µg/ml, 3 min) and propidium iodide (PI, 20 µg/ml, 30 s) and assessed by fluorescence microscopy using a Nikon Eclipse 80i microscope coupled to a DM 1200C digital camera using a 20x objective. For determination of apoptotic cells at the single cell level, cells were plated at about 5×10^4^ cells/ml and incubated with test solutions for 72 h. Apoptotic cells were revealed by the terminal deoxynucleotidyl transferase-mediated dUTP nick-end labeling (TUNEL) method by fluorescence imaging and a cell death detection kit following the protocol provided by the manufacturer.

### Cell ATP levels

Cerebellar granule cells were plated in 4-well plates and cultured with test solutions for 72 h. Then, cells were washed twice with PBS at 37°C and 1 ml of boiling 20 mM Tris, pH, 7.75 and 4 mM EDTA was added. After 2 min, samples were centrifuged for 4 min at 10.000 g. ATP was measured later from the supernatant by the luciferin-luciferase assay using a Cairn photon counting device (Cairn Research, UK) and a standard curve prepared using pure ATP over a 10^−5^ to 10^−9^ M concentration range.

### Statistics

When only two means were compared, Student's t test was used. For more than two groups, statistical significance of the data was assessed by ANOVA and compared using Bonferroni's multiple comparison test. Differences were considered significant at p<0,05.

## Supporting Information

Figure S1 **Characterization of Aβ oligomers and fibrils.** *A*. SDS-PAGE and silver staining of an oligomeric Aβ_1–42_ sample. Arrows point to bands reflecting the presence of monomers, dimers and tetramers. Representative of 3 experiments. *B*, SDS-PAGE and silver staining of a fibrillar Aβ_1–42_ sample. Arrows point to bands reflecting the presence of monomers, dimers, tetramers. Some larger oligomerization species also are apparent in the gel. In addition, a certain amount of large molecular weight fibrils incapable of entering the separating gel is also pointed on top. Representative of 3 experiments. *C*. Electron microscopy was used in order to characterize Aß_1–42_ fibrils. Negative staining using uranyl acetate undoubtedly showed the presence of large fibrils in solution. Most fibrils were similar in width and with a length that usually varied between 200 and 800 nm. Bar represents 200 nm. Representative of 6 experiments. Aβ_1–42_ oligomers and fibrils were also characterized by amino acid analysis for testing actual concentration values and composition (data not shown).
(9.36 MB TIF)

Figure S2 **Aβ_25-35_ induces apoptosis and cell death in cerebellar granule cells.** *A.* Cerebellar granule cells were cultured for 72 h in vehicle (control) or Aβ_25–35_ (20 µM) and apoptosis was tested by TUNEL assay. Pictures show nuclei (blue) and apoptotic cells (purple). Bars show % of apoptotic cells (n = 3; *p<0,05). Scale bar represents 10 µm. *B*. Cerebellar granule cells were cultured for 72 h with vehicle (control) or Aβ_25–35_ (20 µM) and cell death was assessed by staining with FDA (green, living cells) and PI (red, dead cells). Bars show % of dead cells (n = 3; *p<0 05 *vs*. control). Scale bar represents 10 µm.
(6.71 MB TIF)

Figure S3 **FCCP prevents the mitochondrial (but not the cytosolic) Ca^2+^ rise and cytochrome c release induced by Aβ_25–35._** *A.* Cerebellar granule cells expressing mGA were subjected to bioluminescence for [Ca^2+^]_mit_ measurements. FCCP 100 nM inhibits the increase in [Ca^2+^]_mit_ induced by Aβ_25–35_ (20 µM, 17 cells, 3 experiments). After washout of FCCP, Aβ_25–35_ was able to increase [Ca^2+^]_mit_. *B*. Cerebellar granule cells were loaded with fura2/AM and subjected to fluorescence imaging for [Ca^2+^]_cyt_ measurements. FCCP 100 nM failed to inhibit the increase in [Ca^2+^]_cyt_ induced by Aβ_25–35_ (20 µM) (n = 273 cells, 3 experiments). *C*. Immunofluorescence against cytochrome c was assessed by confocal microscopy in cerebellar granule cells treated with vehicle (Control), Aβ_25–35_ 20 µM (Aβ) and 20 µM Aβ_25–35_ + FCCP 100 nM (Aβ+FCCP) for 72 h. Aβ_25–35_ promotes diffusion of cytochrome c that normally shows a punctate staining reflecting mitochondrial location. 100 nM FCCP prevented diffusion of cytochrome C. Scale bar represents 10 µm. *D*. Bars show % of cells showing diffuse staining for cytochrome c (reflecting cytochrome c release). Aβ_25–35_ (20 µM) increases the percent of cells showing diffuse staining an this effect was inhibited by FCCP 100 nM. ^a^p<0,05 vs. control; ^b^p<0,05 vs. Aβ treated cells. Bars are mean±SEM of 3 independent experiments.
(2.27 MB TIF)

Figure S4 **Aβ oligomers induce ROS formation that is prevented by FCCP and R-flurbiprofen.** Cerebellar granule cells were incubated for 4 h with vehicle, Aβ_1–42_ oligomers (500 nM) and oligomers plus FCCP 100 nM or R-flurbiprofen. Then, ROS production was imaged using the ROS-sensitive probe CM-H2DCFDA. Aβ_1–42_ oligomers induced an increase in fluorescence compared to the vehicle that was prevented by FCCP and R-flurbiprofen. The Pictures are representative of 5–9 microscopic fields in at least 3 independent experiments for each condition. Addition of FCCP or R-flurbiprofen alone produced similar results than vehicle (data not shown).
(2.83 MB TIF)

Figure S5 **NSAIDs prevent cytochrome c release and cell death induced by Aβ_25–35._** *A*. Cerebellar granule cells were treated with Aβ_25–35_ (20 µM) for 72 h with or without 1 µM R-flurbiprofen or 100 µM salicylate and fixed for analysis of cytochrome c location using confocal microscopy. Control cells showed a punctate distribution of cytochrome c. Scale bar represents 10 µm. Aβ_25–35_ treated cells show a more diffuse pattern of cytochrome c whereas cells treated with Aβ_25–35_ and R-flurbiprofen show a punctate pattern similar to control cells. Bars show the relative abundance (%) of cells showing diffuse immunostaining in control cells, cells treated with Aβ_25–35_ and cells treated with Aβ_25–35_ plus 1 µM R-flurbiprofen (B) or 100 µM salicylate (C). (*p<0,05 vs. control; #p<0,05 vs. Aβ). *D.* Effects of ibuprofen (Ibu), indomethacin (Indo) and sulindac sulfide (Sul), all tested at 1 µM, on cell death induced by Aβ_25–35_ (20 µM) as assessed by dye exclusion studies. *E*. Effects of R- and S-flurbiprofen (Rf and Sf), both tested at 1 µM, on cell death induced by Aβ_25–35_. *p<0,05 vs. control; ^#^p<0,05 vs. Aβ. All data are representative of 3 experiments.
(2.10 MB TIF)
