# Supplementary figures and images for: Mitochondrial Ca2+ Overload Underlies Aβ Oligomers Neurotoxicity Providing an Unexpected Mechanism of Neuroprotection by NSAIDs
Source: PLoS One. 2008 Jul 23;3(7):e2718. doi: 10.1371/journal.pone.0002718 (PMC2447871; doi:10.1371/journal.pone.0002718)

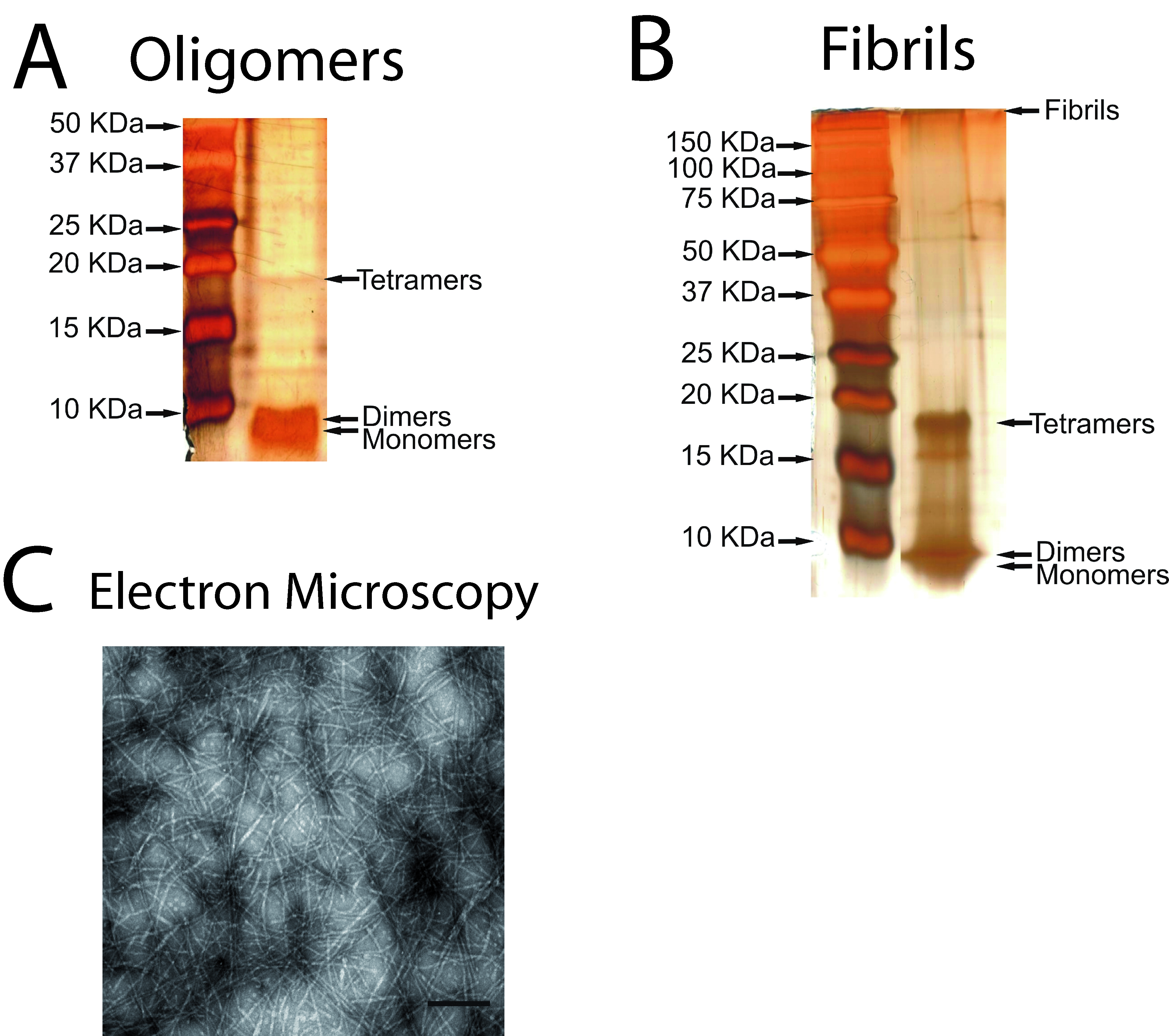

Supplement: Figure S1 — Characterization of Aβ oligomers and fibrils. A. SDS-PAGE and silver staining of an oligomeric Aβ1–42 sample. Arrows point to bands reflecting the presence of monomers, dimers and tetramers. Representative of 3 experiments. B, SDS-PAGE and silver staining of a fibrillar Aβ1–42 sample. Arrows point to bands reflecting the presence of monomers, dimers, tetramers. Some larger oligomerization species also are apparent in the gel. In addition, a certain amount of large molecular weight fibrils incapable of entering the separating gel is also pointed on top. Representative of 3 experiments. C. Electron microscopy was used in order to characterize Aß1–42 fibrils. Negative staining using uranyl acetate undoubtedly showed the presence of large fibrils in solution. Most fibrils were similar in width and with a length that usually varied between 200 and 800 nm. Bar represents 200 nm. Representative of 6 experiments. Aβ1–42 oligomers and fibrils were also characterized by amino acid analysis for testing actual concentration values and composition (data not shown). (9.36 MB TIF) [file pone.0002718.s001.tif]

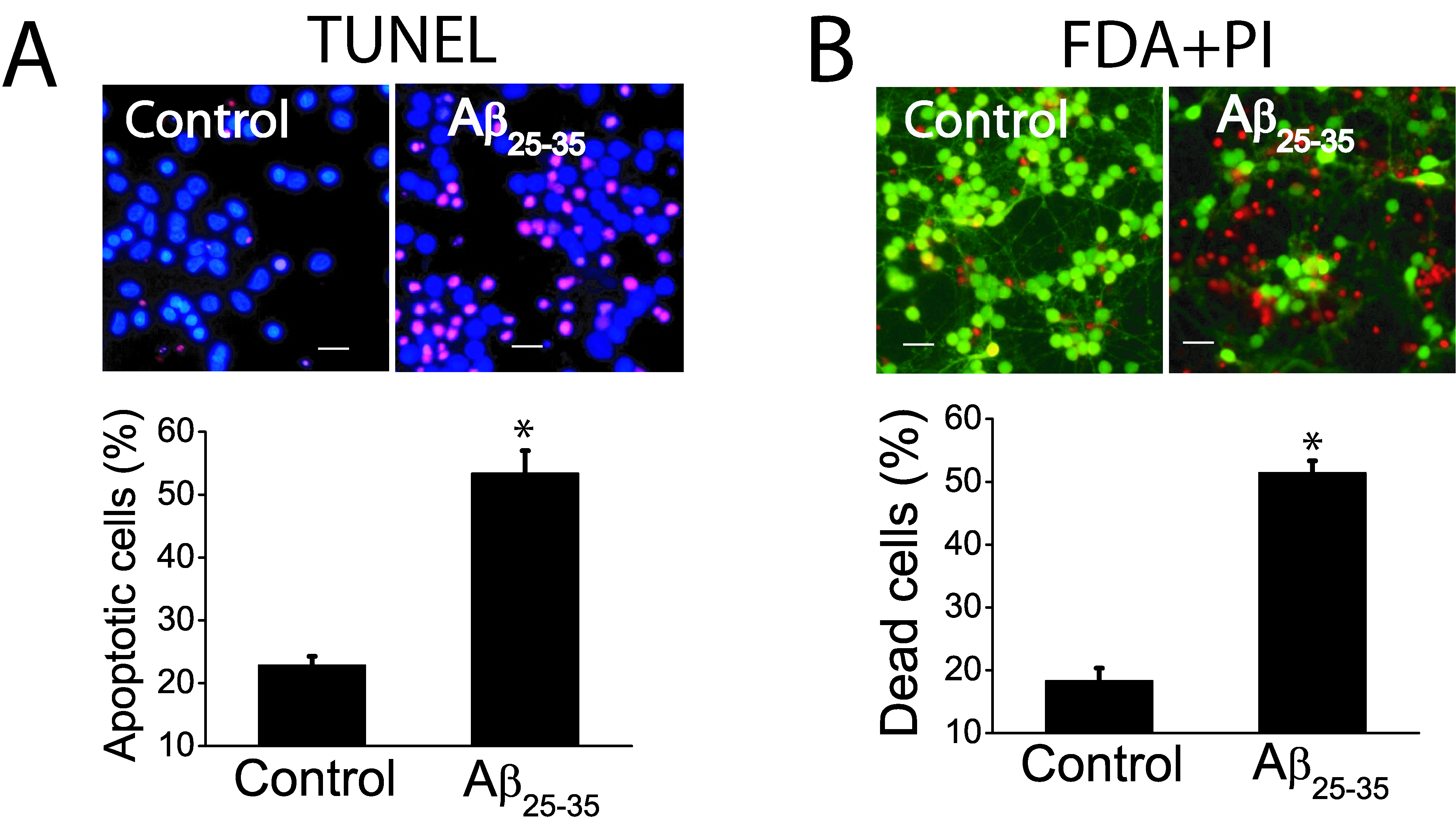

Supplement: Figure S2 — Aβ25-35 induces apoptosis and cell death in cerebellar granule cells. A. Cerebellar granule cells were cultured for 72 h in vehicle (control) or Aβ25–35 (20 µM) and apoptosis was tested by TUNEL assay. Pictures show nuclei (blue) and apoptotic cells (purple). Bars show % of apoptotic cells (n = 3; *p<0,05). Scale bar represents 10 µm. B. Cerebellar granule cells were cultured for 72 h with vehicle (control) or Aβ25–35 (20 µM) and cell death was assessed by staining with FDA (green, living cells) and PI (red, dead cells). Bars show % of dead cells (n = 3; *p<0 05 vs. control). Scale bar represents 10 µm. (6.71 MB TIF) [file pone.0002718.s002.tif]

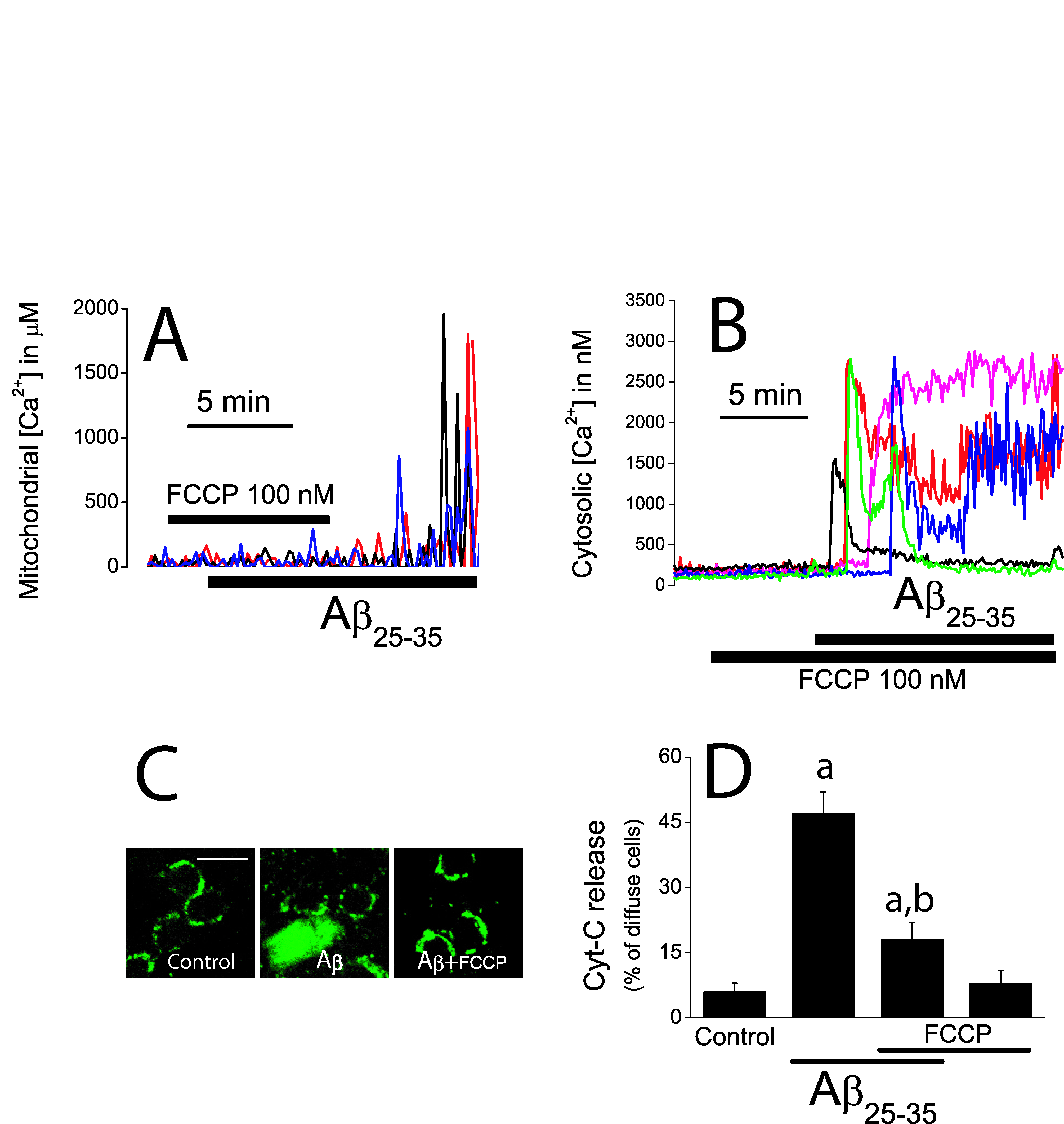

Supplement: Figure S3 — FCCP prevents the mitochondrial (but not the cytosolic) Ca2+ rise and cytochrome c release induced by Aβ25–35. A. Cerebellar granule cells expressing mGA were subjected to bioluminescence for [Ca2+]mit measurements. FCCP 100 nM inhibits the increase in [Ca2+]mit induced by Aβ25–35 (20 µM, 17 cells, 3 experiments). After washout of FCCP, Aβ25–35 was able to increase [Ca2+]mit. B. Cerebellar granule cells were loaded with fura2/AM and subjected to fluorescence imaging for [Ca2+]cyt measurements. FCCP 100 nM failed to inhibit the increase in [Ca2+]cyt induced by Aβ25–35 (20 µM) (n = 273 cells, 3 experiments). C. Immunofluorescence against cytochrome c was assessed by confocal microscopy in cerebellar granule cells treated with vehicle (Control), Aβ25–35 20 µM (Aβ) and 20 µM Aβ25–35 + FCCP 100 nM (Aβ+FCCP) for 72 h. Aβ25–35 promotes diffusion of cytochrome c that normally shows a punctate staining reflecting mitochondrial location. 100 nM FCCP prevented diffusion of cytochrome C. Scale bar represents 10 µm. D. Bars show % of cells showing diffuse staining for cytochrome c (reflecting cytochrome c release). Aβ25–35 (20 µM) increases the percent of cells showing diffuse staining an this effect was inhibited by FCCP 100 nM. ap<0,05 vs. control; bp<0,05 vs. Aβ treated cells. Bars are mean±SEM of 3 independent experiments. (2.27 MB TIF) [file pone.0002718.s003.tif]

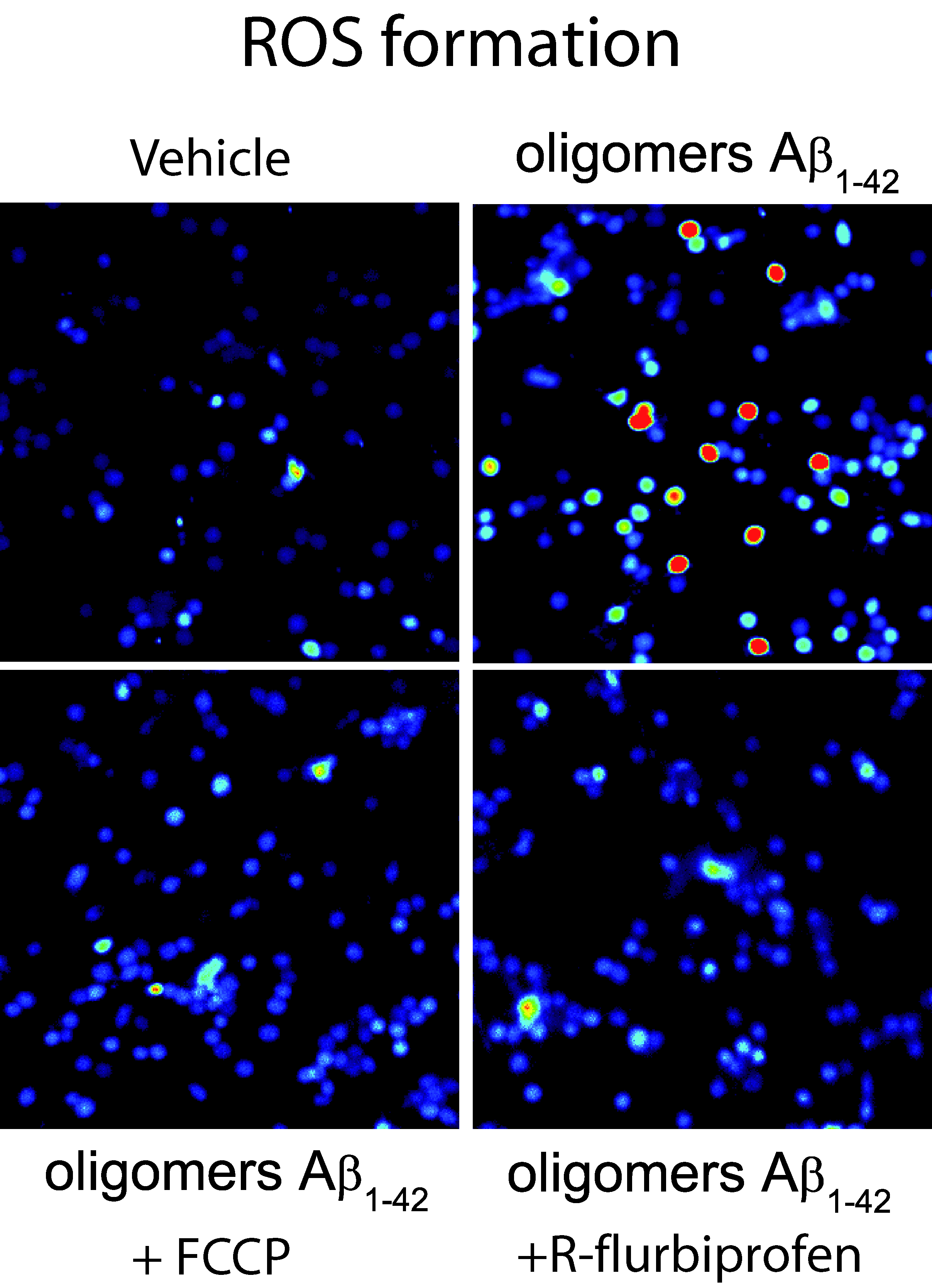

Supplement: Figure S4 — Aβ oligomers induce ROS formation that is prevented by FCCP and R-flurbiprofen. Cerebellar granule cells were incubated for 4 h with vehicle, Aβ1–42 oligomers (500 nM) and oligomers plus FCCP 100 nM or R-flurbiprofen. Then, ROS production was imaged using the ROS-sensitive probe CM-H2DCFDA. Aβ1–42 oligomers induced an increase in fluorescence compared to the vehicle that was prevented by FCCP and R-flurbiprofen. The Pictures are representative of 5–9 microscopic fields in at least 3 independent experiments for each condition. Addition of FCCP or R-flurbiprofen alone produced similar results than vehicle (data not shown). (2.83 MB TIF) [file pone.0002718.s004.tif]

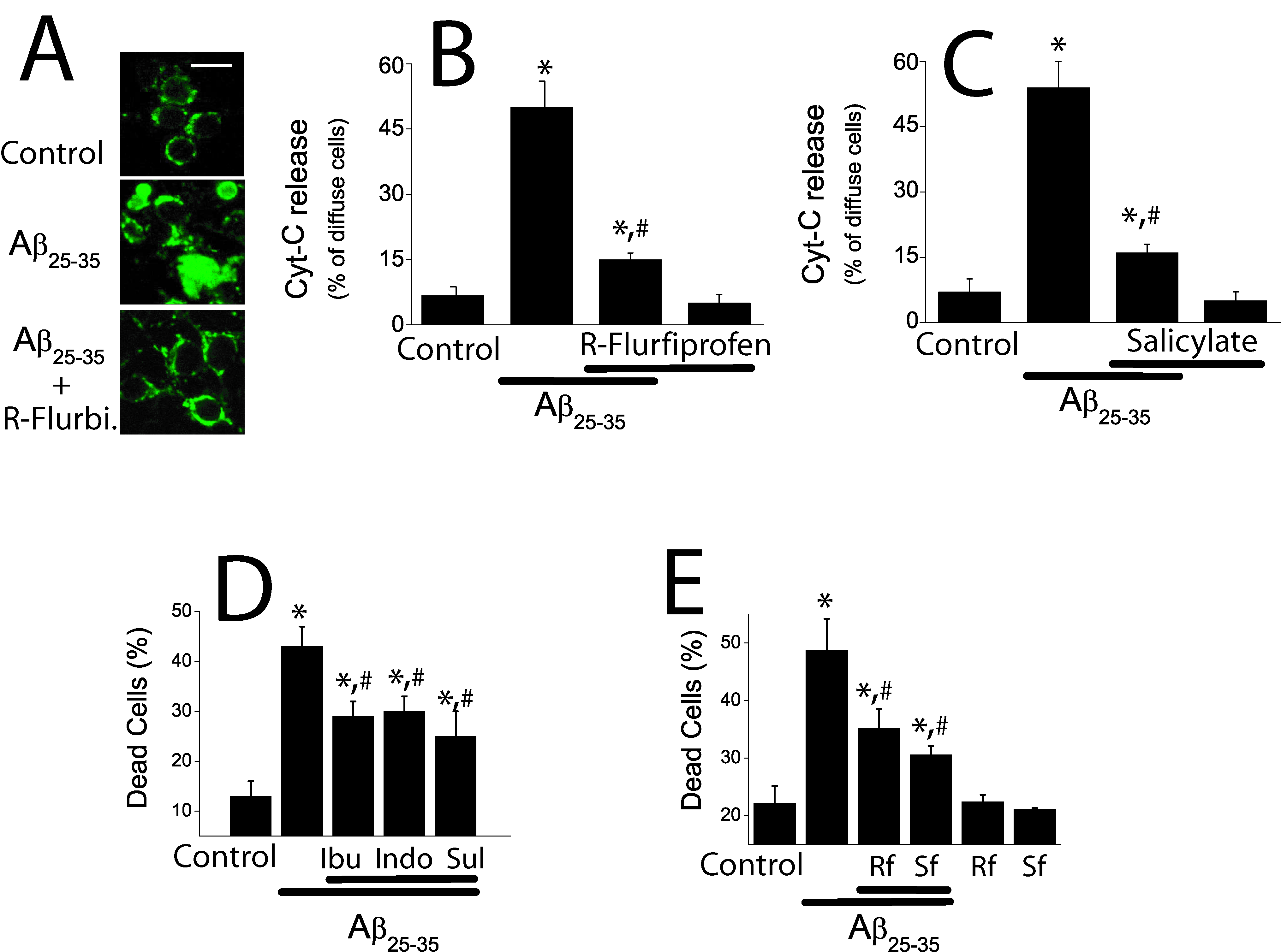

Supplement: Figure S5 — NSAIDs prevent cytochrome c release and cell death induced by Aβ25–35. A. Cerebellar granule cells were treated with Aβ25–35 (20 µM) for 72 h with or without 1 µM R-flurbiprofen or 100 µM salicylate and fixed for analysis of cytochrome c location using confocal microscopy. Control cells showed a punctate distribution of cytochrome c. Scale bar represents 10 µm. Aβ25–35 treated cells show a more diffuse pattern of cytochrome c whereas cells treated with Aβ25–35 and R-flurbiprofen show a punctate pattern similar to control cells. Bars show the relative abundance (%) of cells showing diffuse immunostaining in control cells, cells treated with Aβ25–35 and cells treated with Aβ25–35 plus 1 µM R-flurbiprofen (B) or 100 µM salicylate (C). (*p<0,05 vs. control; #p<0,05 vs. Aβ). D. Effects of ibuprofen (Ibu), indomethacin (Indo) and sulindac sulfide (Sul), all tested at 1 µM, on cell death induced by Aβ25–35 (20 µM) as assessed by dye exclusion studies. E. Effects of R- and S-flurbiprofen (Rf and Sf), both tested at 1 µM, on cell death induced by Aβ25–35. *p<0,05 vs. control; #p<0,05 vs. Aβ. All data are representative of 3 experiments. (2.10 MB TIF) [file pone.0002718.s005.tif]
